# An Immunological Marker of Tolerance to Infection in Wild Rodents

**DOI:** 10.1371/journal.pbio.1001901

**Published:** 2014-07-08

**Authors:** Joseph A. Jackson, Amy J. Hall, Ida M. Friberg, Catriona Ralli, Ann Lowe, Malgorzata Zawadzka, Andrew K. Turner, Alexander Stewart, Richard J. Birtles, Steve Paterson, Janette E. Bradley, Mike Begon

**Affiliations:** 1 IBERS, Aberystwyth University, Aberystwyth, United Kingdom; 2 School of Life Sciences, The University of Nottingham, Nottingham, United Kingdom; 3 Institute of Integrative Biology, The University of Liverpool, Liverpool, United Kingdom; 4 School of Environment and Life Sciences, University of Salford, Salford, United Kingdom; Stanford University, United States of America

## Abstract

Hosts are likely to respond to parasitic infections by a combination of resistance (expulsion of pathogens) and tolerance (active mitigation of pathology). Of these strategies, the basis of tolerance in animal hosts is relatively poorly understood, with especially little known about how tolerance is manifested in natural populations. We monitored a natural population of field voles using longitudinal and cross-sectional sampling modes and taking measurements on body condition, infection, immune gene expression, and survival. Using analyses stratified by life history stage, we demonstrate a pattern of tolerance to macroparasites in mature compared to immature males. In comparison to immature males, mature males resisted infection less and instead increased investment in body condition in response to accumulating burdens, but at the expense of reduced reproductive effort. We identified expression of the transcription factor Gata3 (a mediator of Th2 immunity) as an immunological biomarker of this tolerance response. Time series data for individual animals suggested that macroparasite infections gave rise to increased expression of Gata3, which gave rise to improved body condition and enhanced survival as hosts aged. These findings provide a clear and unexpected insight into tolerance responses (and their life history sequelae) in a natural vertebrate population. The demonstration that such responses (potentially promoting parasite transmission) can move from resistance to tolerance through the course of an individual's lifetime emphasises the need to incorporate them into our understanding of the dynamics and risk of infection in the natural environment. Moreover, the identification of Gata3 as a marker of tolerance to macroparasites raises important new questions regarding the role of Th2 immunity and the mechanistic nature of the tolerance response itself. A more manipulative, experimental approach is likely to be valuable in elaborating this further.

## Abstract 2

A large-scale field study in naturally occurring vole populations identified gene expression changes over time and demonstrates how wild mammals exhibit tolerance to chronic parasite infections.

## Author Summary

Hosts do not always resist parasites. And once infection establishes, relatively little is known of how naturally occurring hosts tolerate (mitigate for) adverse effects, or what the life history consequences of this tolerance may be. In this article we demonstrate a pattern of tolerance to parasitic worms and arthropods in wild voles and link this to increased expression of an immunological biomarker. The biomarker, Gata3, is triggered by infection, precedes significant changes in body condition, and impacts on fecundity and survival. These results point to the considerable ecological importance of tolerance in wild vertebrates and to how poorly it is understood, while at the same time giving a new perspective on the natural function of immune response pathways involving Gata3.

## Introduction

There is increasing recognition that the ability to tolerate a parasite's presence, where the host accepts infection but actively limits the damage caused, may be a crucial host defence strategy [1],[2]. This is distinct from resistance, where the focus is on limiting the infection burden itself [3],[4], though in reality hosts often combine tolerance and resistance. Adopting a tolerance strategy may have major implications for the epidemiology and co-evolutionary dynamics of infectious disease through its effects both on individual hosts and on parasite transmission [5],[6]—for example, leading tolerant individuals to become infectious “super spreaders” [7] or promoting the evolution of higher transmissibility in tolerant populations [8]. However, parasite tolerance has often been neglected in animal and human studies [4],[9], and virtually nothing is known of its manifestation in natural systems. Here we focus on the expression of tolerance in a natural population and attempt to identify immunological processes underlying this and their consequences for important fitness components. In doing so, though, we must first address how tolerance can be defined and interpreted in studies of field systems.

The term “tolerance” is used differently by immunologists and disease ecologists. In moving towards some unification of meaning, we must distinguish between pattern and process. The pattern associated with tolerance, typically the main focus of ecologists, is a relative insensitivity of host health (fitness) to increases in parasite burden [4]. This can result from various host responses (processes) that protect the host from the parasite without reducing parasite fitness. Examples include the up-regulation of wound healing or the down-regulation of immunopathology [9]. The latter would often involve the process conventionally termed tolerance by immunologists: the limitation of T- or B-cell responsiveness to cognate antigens. The pattern of tolerance may also result from host responses that ameliorate pathogen virulence (not tolerance in an immunological vocabulary), for example, by up-regulating lipoproteins that protect against endotoxins or serpins that target bacterial proteases [8]. In using the term tolerance, therefore, it is important to distinguish whether it is the pattern or the process that is being referred to, or both. In natural populations, and also in ecological experiments [4], the pattern will often be known, but the process will not. But equally, in laboratory immunology studies, details of the process may be available, but its consequences in a natural setting (the pattern) may not.

The study of tolerance in field systems, then, is an indispensible complement to studies in the laboratory, and there have been calls for the greater integration of data from natural populations on host fitness, immunological variation, and infection pressure [10],[11]. However, field studies prove challenging in separating pertinent patterns from background noise with other causes and in distinguishing causal relationships from simple associations. Hence, it is necessary to predefine putative markers (“signatures”) of tolerance (patterns) and to assess these within a study framework aimed at disentangling alternative causalities. To achieve this, we draw on the perspective provided by an emphasis on “phase space” [12], where the important distinction is drawn between tolerance curves and phase or disease curves. In tolerance curves, the relationship between host health and parasite load is plotted, with each data point being an observation contributed by a different individual at a standardised stage in an infection (e.g., maximal parasite load). A more tolerant population or subgroup would then be one in which the slope of this relationship was less negative, and a slope of zero (or even more, a positive slope) would be strongly suggestive of tolerance. In phase curves, by contrast, the relationship between an individual host's health and parasite load is plotted, with each data point being an observation at a different stage in the infection. A more tolerant individual would then be one in which the decline in health with increasing load was less steep, and no decline at all (or even more, a positive slope) would be strongly suggestive of tolerance. This phase space perspective also highlights the absence of any agreed measure to quantify health but favours the use of “gross” (whole organism) measures such as those we have adopted here [12].

We hypothesised, first, that different groups of individuals (e.g., life-history stages) will differ in their tolerance—their relationship between health and parasite load—and second, that those individuals that maintain good health in relation to parasite burden (i.e., are more tolerant) will express more of the physiological pattern associated with tolerance. We monitored immunological gene expression, infection, body condition, and survival in replicated natural populations of field voles, *Microtus agrestis*, using interwoven cross-sectional and longitudinal sampling protocols. This hybrid study design took advantage of the greater range and precision of measurement possible in destructive (cross-sectional) sampling, and of the stronger causal inferences possible in capture–recapture (longitudinal) sequences, where driver variables may be observed to precede the responses they trigger [13]. Our objective was to identify patterns of tolerance, to link these to biomarkers among gene expression variables, and then to examine the life history consequences of the tolerance strategy through the surrogate of biomarker expression. Our overall aim was to place tolerance within a life history context and at the same time suggest possible immunological mechanisms (processes) based on the biomarker(s) identified.

Specifically, adopting the phase space perspective (above), plotting our cross-sectional data comes closest to generating a tolerance curve. However, infection stage cannot be standardized in observations on natural populations. Hence, a less negative, zero, or even positive relationship between health and parasite load will be consistent with tolerance (and suggestive of it), but alternative explanations cannot be excluded. By contrast, our longitudinal data generate sequences of time points on a phase curve for a “typical” individual. A pattern in which health fails to decline (or even increases) following an increase in parasite load will therefore be directly indicative of tolerance. The case for demonstrable tolerance will, of course, be strongest if the results from the cross-sectional and longitudinal studies are consistent with one another. In contrast to tolerance, resistance resulting from acquired responses (usually the key component of resistance in co-adapted host–parasite associations; e.g., [14]) is likely to be characterised by a decelerating or even negative accumulation of parasites over time and by a negative association between resistance markers and infection levels.

## Results

### Field Design and Analysis

Our sampling, carried out in natural *M. agrestis* populations in Kielder Forest, Northumberland, United Kingdom, involved a cross-sectional component (*n* = 576 destructively sampled voles) and a longitudinal component (*n* = 920 marked individuals monitored through time, with *n* = 1,665 sampling points) and was replicated at two different sites in each of 2 y. Biometric and infection data were recorded at all sampling points. In the cross-sectional component, ligands for Toll-like receptors (TLRs) 2 and 7 were used to stimulate splenocyte cultures, from which we measured the expression of immune genes involved in regulatory (IL-10, TGF-β1) and effector (IRF5, IL-1β) pathways recruited during antimicrobial pattern recognition responses. In addition, the expression of genes reflecting different T-helper phenotypes was measured in nonspecifically (mitogen, PHA-L) activated splenocytes. The activity of regulatory T cells was represented by the anti-inflammatory cytokines IL-10 and TGF-β1 and the transcription factor FoxP3. The activity of T-helper cell type 1 (Th1) responses was represented by the pro-inflammatory cytokine IFN-γ and transcription factor Tbet and that of Th2 responses by the transcription factor Gata3. In the longitudinal component, constitutive expression of IFN-γ, IL-10, and Gata3 were measured in peripheral blood.

Our overall analytical strategy (initially using standard statistical modelling techniques for single response variables) was to begin with our more detailed cross-sectional data and search first for the patterns of resistance or tolerance described above. We then sought to identify immunological gene expression markers of these patterns and, in turn, to link these to infection and life history variables. Next, we used our independent longitudinal data to corroborate the robustness of the immunological markers, place them within a stronger cause–effect context in relation to the infection and life history variables, and measure their effect on survival. Finally, we returned to the cross-sectional data and used structural equations modelling (SEM) to support the interdependencies between variables indicated by the longitudinal data. In analyses of the cross-sectional data, we primarily represented macroparasite infection using a reduced variable derived as scores from the first principal component of a principal components analysis (PCA) of the most common species that would be expected to be in contact with the host immune system (fleas, ticks, and adult tapeworms) (Table S1). In the search for immunological markers, this variable (PC^M main^) was tested first against similarly reduced immune gene expression variables (Table S2) and then against individual variables using multiplicity adjustments. Equivalent results were obtained if PC^M main^ was replaced in analyses by a similar reduced variable for all macroparasite species recorded (PC^M^) or by measures of some influential species (e.g., ticks). However, the composite measure (PC^M main^) has the advantage that relationships with host responses will not be diluted when, for example, a low burden for one parasite is accompanied by high burdens for others, such that the response to the low burden is “greater than expected,” driven by the other parasites.

### Mature Males Are Less Resistant and More Tolerant

**Figure 1:**
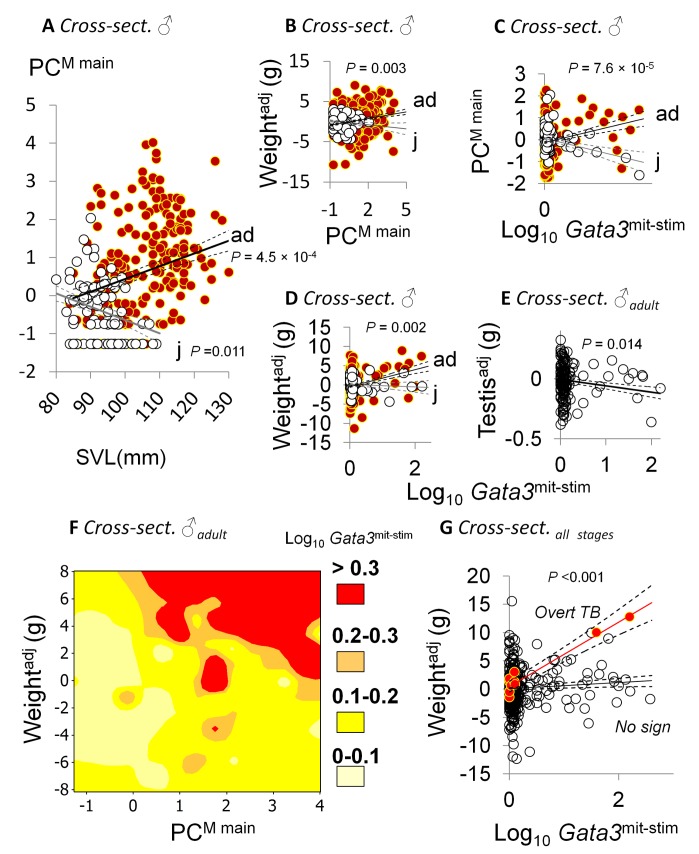
Patterns of tolerance and resistance in stratified analyses, with Gata3 as a biomarker. Cross-sectional study (cross-sect.). (A–D) Differing associations in mature (ad) and immature male voles (j) between principal component scores representing grouped macroparasite infection (main influential species, PC^M main^) and SVL (A), body condition (residual body weight, Weight^adj^) and PC^M main^ (B), PC^M main^ and log-transformed Gata3 expression in nonspecifically stimulated splenocytes (Gata3^mit-stim^) (C), and body condition and log-transformed Gata3^mit-stim^ (D). (E) Association between testis condition (residual testis weight, Testis^adj^) and log-transformed Gata3^mit-stim^ in mature male voles. (F) Contour map representing log-transformed Gata3^mit-stim^ against variation in body condition and PC^M main^ in mature males. (G) Association between body condition and log-transformed Gata3^mit-stim^ in voles with overt TB or with no sign of TB (all stages). (A–E, G) Scatter of points represent actual observations (A) or (for graphical purposes) partial residuals (B–E, G) from LMMs; solid lines are model predictions averaged across all other terms in model; *p* values relate to main effects in LMM analyses conducted separately for mature or immature males (A, E) or Stage×Predictor interactions in LMMs containing different slopes for mature and immature males (B–D); 1 s.e. given above and below model predictions (dashed lines).

In our initial search for patterns of tolerance or acquired resistance, we used general and generalized linear mixed models (LMMs and GLMMs; details in Methods S1). In the overall population (in models with slopes averaged across life history stages), macroparasites (PC^M main^) tended to accumulate linearly with individual size and age, with no indication that acquired resistance decelerated the acquisition of infection in older animals (Figure S5). This was not uniform across life history stages, though, with a particularly robust contrast occurring between mature males (those with large descended testes and expanded seminal vesicles) and immature males (Figure 1A). Mature males accumulated macroparasites linearly as age indicators increased (SVL [mm], slope parameter 0.034±0.009, *p* = 4.5×10^−4^; eye lens weight [g], 283±79, *p* = 3.9×10^−4^), but immature males supported less infection as age indicators increased (SVL, −0.035±0.013, *p* = .011; lens weight, −317±97, *p* = .002). This pattern is consistent with resistance building up prior to maturity but being absent postmaturity (Figure 1A).

By contrast, there was a strong overall pattern consistent with a tolerance response. To examine this, we used as a proxy for individual health, not simple measures of weight but “body condition”: body (or organ) weight normalised for individual size – snout-vent length (SVL) and its quadratic term (see Methods S1). Considering all voles together, those in the best condition tended to be infected with the most macroparasites (body condition, *p*<5×10^−7^; Tables S3, S4, S5). Furthermore, stage-specific differences in tolerance were suggested by heterogenous slopes of body condition on macroparasite loads (PC^M main^) amongst different life history stages. A particularly robust contrast occurred again between mature males, which showed a strong positive relationship between parasite burden and body condition, and immature males, which showed no such relationship (Figure 1B, LMM, Stage×PC^M main^ interaction, *p* = .003) (Table S7). Amongst the microparasitic infections that we surveyed, animals displaying overt signs of tuberculosis (TB) (*Mycobacterium microti*) were also in better condition relative to others (body condition, *p* = .018; Tables S3, S4).

### Gata3 as a Biomarker for Tolerance in Mature Males

To identify immunological markers for a tolerance strategy and determine their consequences for host fitness, we then focused primarily on the dichotomy between mature and immature males, as the former showed the strongest increased body condition response to macroparasite infection but no epidemiological evidence of resistance. Patterns in females, which may be obscured by immunosuppression during pregnancy, were less clear cut, as discussed briefly below.

We began by asking (using LMM analyses) whether the trend for apparent tolerance rather than resistance in mature versus immature males was linked to variation in immune gene expression. We found that a single parameter, mitogen-stimulated Gata3 expression in cultured splenocytes (Gata3^mit-stim^), was consistently and strongly associated with macroparasite burden, host condition, and life history in mature males and also showed a different pattern in immature males. First, Gata3^mit-stim^ correlated with macroparasite burden positively in mature males (Figure 1C, *p* = .007; Table S11) and negatively in immature males (Figure 1C). Second, Gata3^mit-stim^ was positively correlated with liver and body condition in mature males (Figure 1D, *p* = .027 and 7.1×10^−5^, respectively; Tables S8 and S9) but not immature males (Figure 1D), even after controlling for the association between macroparasite burden and body condition noted above. Third, Gata3^mit-stim^ had a significant negative association with size-adjusted testis weight (an estimator of male reproductive effort) in mature males (Figure 1E, *p* = .014; Table S9). Thus, Gata3 expression was higher in mature males in good condition and infected with many macroparasites (Figure 1F) but with proportionately smaller reproductive investment. Notably, Gata3^mit-stim^ expression was also a strong marker for elevated condition among animals with overt TB (Figure 1G, *p*<.001; Table S10).

Moreover, because Gata3 would usually be involved where Th2 immunity mediates resistance to macroparasites [15], the differing stage associations of macroparasites with Gata3 expression observed (Figure 1C) are indicative of changes in resistance. Thus, the negative association in immature males contrasted with the positive association in mature males is further evidence (additional to the stage-specific associations of infection with age indicators) (Figure 1A) that immature males may resist macroparasite infections, whereas mature males do not.

Gata3 expression, then, may be a useful biomarker for either resistance or tolerance where a predominant pattern of one or the other can be established within groups, as it can in males. Nonpregnant females showed similar tolerance-like accumulations of macroparasites with age indicators and also increases in condition at higher macroparasite burdens. However, *post hoc* analyses indicated that these trends were not clearly marked by splenic Gata3 expression. This might be because of the re-adjustment of T-helper cell responses often seen during mammalian pregnancy [16] and also because among nonmating nonpregnant females (the majority of the female sample) prereproductives and those returning to a nonmating (imperforate) state following pregnancy could not be distinguished. Females also showed much lower infection levels than males (Figure S5B), and older females in mating condition, which were most heavily parasitized (Figure S5B) and thus most likely to show clear tolerance responses, were poorly represented in our cross-sectional sample (*n* = 47).

### Dynamics of Gata3 Expression in the Tolerance Response

**Figure 2:**
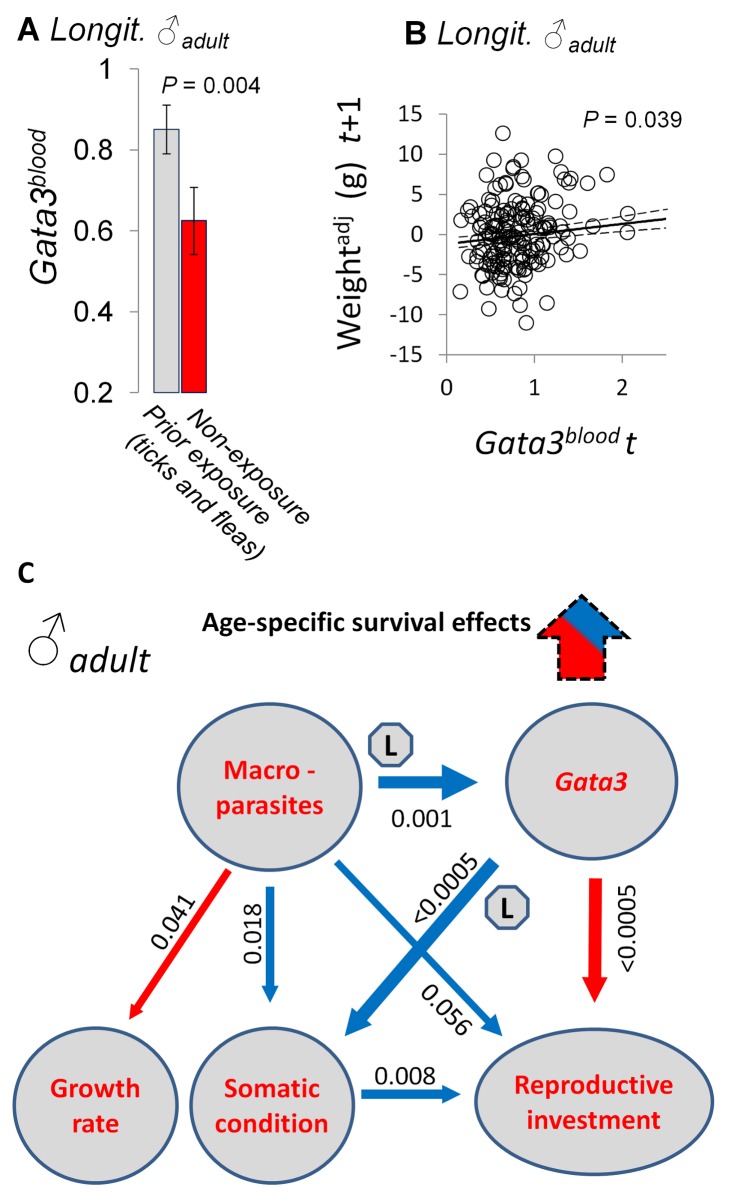
The dynamics of Gata3 expression in tolerance responses. (A) Longitudinal study (Longit.). A history of exposure to ticks or fleas at previous sampling points was associated with higher levels of peripheral blood Gata3 (Gata3^blood^) expression; LMM prediction with 1 s.e. above and below. (B) Longitudinal study. Positive association of Gata3 expression (Gata3^blood^) in peripheral blood with weight increase (adjusted for starting weight) in the subsequent month. Scatter of points represents final weight residuals on starting weight; solid line is an LMM prediction averaged across all other terms in model; 1 s.e. given above and below model prediction (dashed lines). (C) A causal hypothesis refined using SEM of data for mature males in the cross-sectional study. Where the flow of causality (solid arrow directions) depicted in the set of relationships was supported by temporal analyses in the longitudinal study, this is indicated with an “L”. Details of analysis in Methods S1. The *p* values for respective parameters in the SEM are given alongside arrows (whose widths are proportional to the *z* score). Blue arrows indicate a positive effect and red arrows a negative effect. The arrow with a dashed margin indicates that age-specific effects on survival were additionally found for Gata3 expression (in blood) in separate analyses of the longitudinal data (not part of the SEM).

Gata3 is a pivotal transcription factor involved in the development of Th2 cells [17]–[19], and its expression in mitogen-stimulated splenocyte cultures is likely to derive from proliferated Th cell populations. Th2 responses are typically triggered by macroparasite infection [20]–[24]. Hence, an initial hypothesis was that macroparasites stimulated higher Gata3 expression in mature males and that either macroparasites or Gata3 expression might then affect host condition and reproductive effort. To further explore possible causal links, we turned to our longitudinal data, focusing on macroparasites. In mature males, exposure to common blood-feeding ectoparasites was strongly associated with increased constitutive Gata3 expression in peripheral blood (Gata3^blood^) in the following month (LMM, Figure 2A, *p* = .010; Table S12). (By contrast, the instantaneous association tended to be negative, perhaps reflecting some level of resistance to ectoparasites due to peripheral blood responses.) In turn, Gata3^blood^ was positively associated with weight gain (adjusted for starting body weight) in the following month (LMM, Figure 2B, *p* = .039; Table S13). (By contrast, it was unrelated to weight gain in the preceding month.) Taken together, therefore, the longitudinal data suggest that in mature males at least, macroparasites are the driver of high Gata3 expression, which in turn is part of a response that promotes elevated body condition. That is, as individuals progress along their phase (disease) curve [12], one proxy for health (body condition), far from declining as parasite load increases, appears to increase, indicative of a tolerance response to infection.

In applying SEM to our cross-sectional data (Figure 2C; Tables S14 and S15), we specifically sought the SEM that best fitted the data or was indistinguishable from the best in explanatory power, but that was also supported by the details of the longitudinal data analysis above. In fact, the best and one near-best SEM did have this support, though they differed as to whether male reproductive effort had a negative effect on Gata3 expression and body condition or was negatively affected by them (which we take to be more likely biologically). The SEM analysis was thus consistent with a causal path: macroparasites→elevated Gata3→increased somatic condition/reduced reproductive effort. As a final *post hoc* step in the sequence of analyses in the cross-sectional data, we reiterated the SEM, replacing Gata3^mit-stim^ with each of the other immune gene expression variables in turn. No other variable approached the significant configuration of coefficients seen for Gata3^mit-stim^, suggesting that this response in particular (and not a wider combination of the expression responses measured) was linked to the tolerance pattern.

### Gata3 Expression Predicts Age-Specific Survivorship

**Figure 3:**
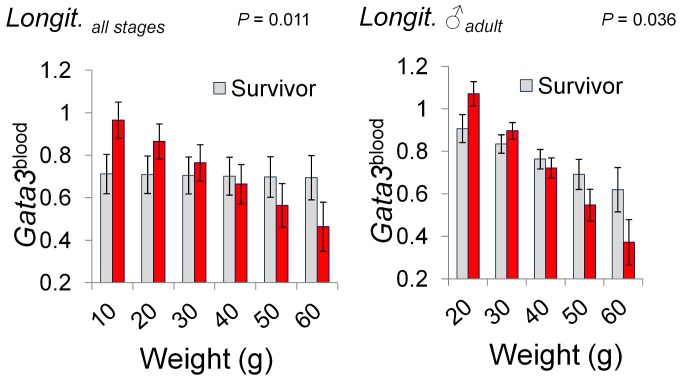
Association of Gata3 expression with survivorship. Longitudinal study (Longit.). In both the full sample and in mature males, analyses of return rates indicated that higher Gata3^blood^ expression was associated with lower survival in smaller animals and higher survival in the largest animals. Predictions from LMMs (for details see Methods S1); 1 s.e. given above and below model predictions.

Constitutive Gata3 expression in peripheral blood (Gata3^blood^), which decreased with age (using weight as a surrogate), was a significant predictor of survivorship in the longitudinal data (Figure 3; Tables S16 and S17). This effect occurred both for adult males and in the overall dataset including all stages (adult males comprising 27% of records in the longitudinal study). In both the overall and the adult male analyses, younger animals expressing high Gata3^blood^ had lower survival, but older animals expressing high Gata3^blood^ had higher survival (GLMM, Figure 3; Gata3^blood^×Weight interaction in mature males, *p* = .036; in all stages, *p* = .011).

## Discussion

These analyses suggest that expression of Gata3 (a master transcription factor involved in the differentiation of Th2 cells) can, in some individuals, be a marker for a physiological programme of tolerance to macroparasite infection, with implications for body condition, fecundity, and survival. Tolerance involved apparent overcompensation in body condition following infection, but accompanying that was downwards readjustment of fecundity, and age-dependent changes in survival, with survival improving in older tolerant (Gata3^hi^) animals. Our longitudinal analyses suggest, moreover, that rather than being a mere correlate, elevated Gata3 expression was triggered by macroparasite infection and preceded the life history readjustments. Some corroborative patterns were also seen for another chronic infectious disease, TB, with animals being in better condition if showing overt signs of infection and especially if also expressing high levels of Gata3.

Our ability to infer tolerance in a complex field situation was facilitated by differing responses, in our cross-sectional study, between immature and mature males. These responses indicated a predominant pattern of resistance to macroparasites in the former and tolerance to macroparasites in the latter. Thus, as immature males aged, their infections reduced, consistent with the development of acquired resistance; also, mitogen-stimulated splenocyte Gata3 expression (Gata3^mit-stim^) was inversely associated with macroparasite infection in these animals. This presents a familiar scenario, consistent with the paradigmatic role of acquired Th2 responses in resistance against macroparasites. Mature males, on the other hand, showed a very different and unexpected pattern. They accumulated macroparasites with age (resulting in very high infection levels) and showed a positive association between Gata3^mit-stim^ and infection. Furthermore, body condition increased in heavily infected animals, whereas Gata3^mit-stim^ was also positively associated with body condition (trends not found in immature males) and negatively associated with male fecundity (adjusted testis weight). Our longitudinal time series data for recaptured adult males indicated that peripheral blood Gata3 expression was triggered by macroparasite exposures and preceded host responses that increased somatic maintenance and ultimately survival (in the oldest Gata3^hi^ animals). This is suggestive of the *process*, and not just the pattern, of tolerance, as the increase in body condition associated with macroparasite infection and Gata3 expression is indicative of a protective response that does not affect the parasite directly.

Gata3 expression in Th2 cells, then, at least in males where there is not the complicating factor of pregnancy, may serve as a biomarker for either resistance or tolerance to macroparasites, depending on whether one of these patterns dominates within a particular group of animals. Further studies are required to determine the relevance of these findings in other vertebrate systems, although the basic data required to establish the context of tolerance or resistance (i.e., indicators of individual age, condition, and infection levels) are well within the routine scope of ecological researchers focussing on infectious disease. As considered further below, the dual aspect of Gata3 expression (marking either tolerance or resistance in different groups of animals) is biologically plausible given the known role of Th2 responses in some pathways leading to resistance to macroparasites [15] but also in pathways promoting wound-healing and tolerance processes [25]. This suggests the possibility, though, that upstream Th2 signals might drive different response mechanisms during resistance and tolerance.

There are alternative possible explanations for some of the cross-sectional associations we observed within mature males. One would be “differential mortality”—that is, high parasite burdens eliciting high Th2 responses, but before our observations are made, animals in poor condition with many macroparasites suffering increased mortality, so that the only animals with high parasite burdens left to observe are those in good condition. A second explanation would be “correlated risk”—that is, increased risk in high condition animals of macroparasite infections, and the Th2 responses they trigger, due, for example, to animals that forage more actively acquiring both more food and more parasites. However, whereas the temporal sequences seen in the longitudinal data directly support the tolerance hypothesis (macroparasites→Gata3→good condition), they support neither differential mortality nor correlated risk, as high Gata3 expression would in neither case precede good condition. Similarly, both differential mortality and correlated risk would predict associations between Gata3 expression and survival that did not change with age, with the former specifically predicting an uncomplicated association of high Gata3 with low survival and poor condition. These predictions, too, are contradicted by our results. Furthermore, differential mortality and correlated risk do not provide explanations for differing cross-sectional associations between Gata3^mit-stim^, body condition, and macroparasite infection in mature and immature males. Hence, we can exclude these alternatives.

Beyond our own system, these results may also provide some general insight into the nature of tolerance, as both pattern and process. The regulatory component of the immune system has often been seen as a potential driver of tolerance, through its role in suppressing immunopathological effector responses. Indeed, helminth parasites have been suggested to increase tolerance and reduce resistance by the elevation of regulatory responses [4]. However, while acknowledging the potential limitations of gene expression data [26], the prominent signature in our study of Gata3 in tolerant animals, rather than of archetypal regulatory genes (IL-10, TGF-β1, FoxP3), suggests that tolerance may in fact be significantly associated with a Th2 mechanism.

In apparent opposition to this, Th2 responses have often been considered to mediate resistance to macroparasites (as in immature males in this study). In the laboratory they may do so via a diverse cascade of effector mechanisms [15]. However, not all effectors necessarily produce resistance to all species [15],[27], and vigorous effector responses have been observed without efficient resistance [27],[28]. Recently, moreover, Th2 responses have also been linked [25] with tolerance to macroparasites. A Th2 environment is well known to promote damage repair mechanisms, including the differentiation of alternatively activated macrophages [29],[30]. Also, generic Th2-associated effectors (e.g., eosinophils, mast cells, antibodies) or Th2-driven fibrotic or granulomatous reactions [25], linked to the expression of Gata3, may mediate the pattern of tolerance by physically containing or more generally limiting the direct pathogenic activities of parasites. Furthermore, Th2 responses in chronic *Mycobacterium* infections have been thought to facilitate clinical disease progression [31]–[33], but the possibility that they function as a tolerance response has not been considered. And finally, although the responses of nonimmunological cell populations were not measured, Gata3 could also be involved in redirecting resources between life history traits during infection, through its role in the differentiation of cells involved in energy stores (e.g., adipocytes [34],[35]) and reproductive investment (e.g., mammary epithelia [36]).

It seems possible that increased body condition and reduced fecundity, in mature males but not immature males, might be adaptive by increasing residual reproductive value during macroparasite infection. This is consistent with a positive influence of Gata3 expression on survival in the oldest males. The contrasting patterns seen between mature and immature males suggest these may face very different cost/benefit scenarios when deploying immune responses against macroparasites. At present we can only speculate about the nature of these pressures, although a number of interesting possibilities might be considered. For example, altered exposure (perhaps elevated in mature males due to increased ranging and social contacts) might raise the immunopathological or energetic costs of resisting infection above thresholds of sustainability. Mature males also, due to their greater prior opportunity to develop robust adaptive immunity to common pathogens, may face fewer consequences from secondarily adopting a more tolerant phenotype. Thus, tolerance processes might have more serious indirect consequences for younger individuals through the inhibition of inflammatory responses against primary microbial infections.

These are the first results to indicate a mechanism of tolerance in a naturally occurring mammalian population and to place this within a holistic framework of host fitness: context-dependent cost–benefit outcomes for individual condition, reproductive investment, and survival. This work therefore illustrates how immunological studies in natural populations may not only benefit from the many advances made in the laboratory but might also feed insights back into mainstream immunology. The results support the view that Th2 responses may play a crucial role in the tolerance of infection, and they more generally add to the growing body of evidence arguing for an increased focus on tolerance in our quest to understand host responses to parasitic infection. Further progress is likely to be facilitated by adopting approaches that incorporate experimental manipulations.

## Materials and Methods

All procedures were carried out under UK Home Office licence regulations. We studied field voles (*M. agrestis*) in Kielder Forest, Northumberland, United Kingdom, using live-trapping to access individual animals from natural populations. Our study was designed to permit the analysis of individual variation in condition and survival, infection status, and the expression of immune genes (additional details in Methods S1). In order to ensure representativeness, we repeated our field design at two spatially separate sites in 2008–2009 and a further two separate sites in 2009–2010 (Figure S1). The study was divided into longitudinal and cross-sectional components. Each site contained a live-trapping grid (∼0.375 ha) of 150 (10×15) regularly spaced traps (3–5 m intervals) placed in optimal habitat (Figure S1). Animals from this grid were marked with passive radio frequency transponders (AVID) and monitored over time, as sequences of capture and recaptures, forming the longitudinal component of the study. At each capture, biometric, infection, and immune expression measurements were taken (Figure S2). On each site there were also satellite transects (with traps spaced at ≥5 m intervals) from which 10 animals per month per site were sampled destructively, forming the basis for the cross-sectional component of the study. Animals from this part of the study were returned to the laboratory, where it was possible to collect a more comprehensive and detailed set of biometric, infection, and immune expression measurements (Figure S2). The transects aimed to sample a very large area of the habitat, providing data representative of the entire population at each site, while at the same time avoiding significant demographic readjustments.

Each site was monitored by monthly trapping sessions between February (in the 2008–2009 season) or April (in the 2009–2010 season) and November, during which the capture–recapture study was carried out on the grid and destructive samples were retained from the transects (Figure S3). At each site, in November at the end of the field season and again in the following March, larger numbers of animals were destructively sampled both from the transects and from the grid habitats, including some animals previously marked with AVID transponders and processed for small tail-tip blood samples as part of the capture–recapture study. These samples also contributed to the overall cross-sectional component of the study (Figure S3).

Measurements of gene expression (IFN-γ, Tbet, IL-2, Gata3, IRF5, IL-10, TGF-β1, FoxP3) in the cross-sectional element of the study focussed on cultured splenocytes and are described in detail in Jackson et al (2011) [20]. Through the combination of stimulatory conditions applied and the genes investigated, these measurements were intended to reflect both innate immune responses (including TLR-mediated responses) and adaptive responses (including Th1, Th2, and T-regulatory responses). In the longitudinal study we measured *in vivo* expression of a subset (IFN-γ, Gata3, IL-10) of the genes investigated in the cross-sectional study, in peripheral blood samples.

Using direct counts or semiquantitative abundance indices, we quantified 25 species of macroparasite in our study animals, some of which were aggregated into ecologically and phylogenetically coherent groups to facilitate analysis (additional details in Methods S1). We also recorded overt symptoms of vole TB caused by *M. microti* and carried out PCR diagnostics for *Bartonella* spp. and *Babesia microti* using previously established methods (see Methods S1, Table S18).

Data were analyzed using LMMs or GLMMs to relate individual variables to potential explanatory variables while allowing for nonindependence due to sampling and immunological assaying structure. SEM was used to assess patterns of interdependency among multiple variables simultaneously. Some variables used in these analyses were principal component scores (reduced from larger sets of partially redundant measurements using PCA). In the case of macroparasites, we used the first principal component (PC^M main^) from a PCA of common taxa likely to be in strong contact with the immune system (i.e., feeding on, or dwelling in or on blood or internal tissues; >20% prevalence overall) as the main analytical variable (see Methods S1 for more details). This component was dominated by high loadings of the same sign for the main macroparasite groups (fleas, ticks, and adult cestodes). Analyses of vole return rates and survivorship were carried out using GLMMs and Cormack-Jolly-Seber (CJS) methods with individual covariates.

In order to control the type 1 error rate, the analyses proceeded according to an *a priori* sequential strategy, starting with a sparing number of initial main hypotheses and moving onto subsequent main hypotheses conditional upon significant results in previous rounds of testing. This strategy also made use of reduced immunological and parasitological data and multiplicity adjustments within each round of main hypothesis tests. More expansive *post hoc* analysis of individual variables and subsets of the data, for the purposes of corroboration and describing biological patterns in more detail, was conducted subsequent to each round of main hypothesis tests. More details on the methods and strategy for data analysis are given in Methods S1 (see Figure S4 and Table S19). Data available from the Dryad Digital Repository: http://dx.doi.org/10.5061/dryad.bk537 [37].

## Supporting Information

Figure S1 Study sites.
(TIF)

Figure S2 Sampling and measurements. Design replicated on two sites in 2008–2009 and two different sites in 2009–2010. Processing steps indicated by solid arrows and measurements by dashed arrows. Gene expression measurements shown in red. For timeline, see Figure S3.
(TIF)

Figure S3 Timeline of sampling.
(TIF)

Figure S4 Flow of main analysis. In order to avoid problems of interpretation due to multiplicity of tests, we carried out our analyses in a stepwise fashion, with each continuation step conditional on significant results in a single or small number of main hypothesis tests in the previous step. For parasite and immune expression data, these main tests initially used reduced variables (principal component, PC, scores). Significant main tests involving PC variables were followed by exhaustive *post hoc* testing of individual variables. Arrows indicate steps in the analysis associated with a particular hypothesis test or tests. Blue arrows indicate steps using data from the cross-sectional study (from which a wider range of more precise immunological, biometric, and parasitological measurements was available). Where a particular individual variable or stratum of hosts was strongly implicated at a given step of the analysis and is focussed upon in the subsequent analysis, this is indicated in red to the right. Yellow arrows indicate steps using longitudinal data (where temporal sequences allowed stronger inference on cause and effect and the estimation of survival). Details of the respective analysis steps are provided in the figures and tables indicated on the left of each arrow (in grey) and the analytical methods employed are indicated on the right (in black) (PCA, principal components analysis; LMM, linear mixed model; GLMM, generalized linear mixed model; SEM, structural equations modelling; CJS, Cormack-Jolly-Seber survival analysis).
(TIF)

Figure S5 Overall associations between macroparasite abundance and host age indicators and life history stage. (A) Associations between parasite abundance and host size and age indicators, averaging slopes across life history groups. Plotted relationships are predictions (±1 standard error) from LMMs of the form: Parasite variable = Process day+LH+SVL (random component: Year×Sampling Point×Site). A quadratic term for SVL was initially included in models, and equivalent models were also analysed with lens weight terms replacing the SVL terms (with similar results). There were highly significant positive linear associations with the principal component representing key macroparasite species, PC^M main^ (see Table S1), and with the individual key macroparasite taxa (small fleas, ticks, and adult cestodes). There were no significant decelerating quadratic terms for SVL or lens weight. The key taxa were common species (>20% prevalence) in strong contact with the host immune system due to feeding habit or definitive site (see Table S1). The common detritivorous ectoparasitic mites (not expected to be in strong contact with the host immune system and perhaps better considered commensals rather than parasites) were significantly negatively linearly associated with SVL and lens weight. This might be related to increased grooming efficiency in older animals or to nidicolous transmission foci. PC^M^, the principal component reflecting variation in the abundance of all macroparasites, strongly contrasted the abundances of key influential species (with positive loadings) with the abundances of common detritivorous mites (with negative loadings) and was thus also positively linearly associated with SVL and lens weight. There was thus no evidence of ongoing buildups of acquired resistance to the key macroparasitic throughout the lives of individuals, although analyses stratified by life history stage, with different macroparasite on age indicator slopes, indicated that resistance might build up in immature males but then collapse postmaturity (Figure 1A, main text). (B) Predicted variation in PC^M main^ with host life history stage from the above model. Estimates ±1 standard error. Infection with the key macroparasitic taxa was concentrated in mating males (with descended testes and expanded seminal vesicles). Mature males carry a much heavier macroparasite load than other life history stages.
(TIF)

Table S1 Multivariate reduction of macroparasite data (cross-sectional study). PCA was used to generate single composite variables (first principal components, PC1) representing the main axis of covariation amongst sets of macroparasite variables. Scores from these PC1 were then used in subsequent analyses to represent “grouped” macroparasite variation. An initial PCA included all of the macroparasite variables. PC1 (designated PC^M^) was dominated by larger loadings for the more common species. These loadings strongly contrasted laelapid and listrophorid mite abundances with those of small fleas, ticks, and intestinal cestodes. A second PCA was carried out including only common macroparasite species that feed directly on host tissues (small fleas and ticks) or that have an intimate association with host mucosal surfaces and adsorb soluble nutrients utilizable by the host (adult cestodes). This excluded all rare species (prevalence <20%). It also excluded common species that do not feed directly on host tissues, do not have an intimate association with host mucosal surfaces, and do not adsorb soluble nutrients. This was the case for listrophorid and laelapid mites, which are primarily deritivores, and pinworms (*Syphacia*), which occur free in the lumen of the colon and feed on bacteria. (Some of these species, especially the detritivorous mites, might better be considered as commensals rather than genuine parasites under normal conditions.) This second PCA produced a very dominant first component (PC^M main^) with large loadings of similar magnitude and the same sign for small fleas, ticks, and adult cestodes, indicating a pattern of general positive covariation among these. In subsequent analyses, PC^M main^ was used as the primary index of macroparasite infection pressure (due to its strong representation of the species likely to be most influential), but PC^M^ was also analyzed secondarily (with similar results).
(DOC)

Table S2 Multivariate reduction of immune expression data (cross-sectional study). PCA was used to reduce sets of immune expression variables into smaller sets of nonredundant composite variables (principal components, PC) reflecting major axes of covariation among the starting variables. Scores from these PCs were then used in subsequent analyses to represent grouped immune responses (i.e., reflecting the major patterns of covariation). Immune expression data were considered in two different ways: (1) expression under stimulatory conditions relative to a calibrator sample (RQ values) and (2) expression indexed to a corresponding unstimulated control culture. Each of these sets of variables was reduced separately, with distinct analyses being carried out within each set for data from TLR-stimulated and mitogen-stimulated cultures. This gave four separate PCAs, from each of which we considered the two largest components (PC1 and PC2) in further analyses. The components were designated: PC1^tlr-stim^ and PC2^tlr-stim^ (respectively, PC1 and PC2 for nonindexed relative expression data from TLR-stimulated cultures); PC1^tlr-index^ and PC2^tlr-index^ (respectively, PC1 and PC2 for indexed expression data from TLR-stimulated cultures); PC1^mit-stim^ and PC2^mit-stim^ (respectively, PC1 and PC2 for nonindexed relative expression data from mitogen-stimulated cultures); and PC1^mit-index^ and PC2^mit-index^ (respectively, PC1 and PC2 for indexed expression data from mitogen-stimulated cultures).
(DOC)

Table S3 Association of body condition with parasitic infections across all host stages (cross-sectional study). Body condition was represented in LMMs by body weight (the response) adjusted for covariates SVL and its quadratic term, initially averaging slopes for these across life history stages. Models considered all of the sampled stages and were of the form: Body weight = LH+Process group+SVL+SVL^2^+LH.SVL+LH.SVL^2^+Parasite variable (random term = Year×Sampling Point×Site). Both of the macroparasite principal component variables (PC^M^ and PC^M main^), several of the individual macroparasite variables, and presence/absence of overt TB all showed significant positive associations with body condition. The only negative association was for Listrophoridae (fur mites), which may possibly have been due to an association between poor condition and compromised grooming. Significant positive association in the main hypothesis test is highlighted in orange; significant associations in *post hoc* tests are highlighted in yellow (positive associations) or grey (negative associations).
(DOC)

Table S4 Association of liver condition with parasitic infections across all host stages (cross-sectional study). Liver condition was represented in LMMs by liver weight (the response) adjusted for covariates SVL and its quadratic term, initially averaging slopes for these across life history stages. Models considered all stages sampled and were of the form: Liver = LH+Process group+SVL+SVL^2^+LH.SVL+LH.SVL^2^+Parasite variable (random term = Year×Sampling Point×Site). Both of the macroparasite principal component variables (PC^M^ and PC^M main^), two of the individual macroparasite variables, and presence/absence of overt TB all showed significant (or marginally nonsignificant) positive associations with liver condition. The only significant negative association was for ear mites, which may possibly have been due to an association between poor condition and compromised grooming. Significant positive association in main hypothesis test is highlighted in orange; significant (or marginally nonsignificant) associations in *post hoc* tests are highlighted in yellow (positive associations) or grey (negative associations).
(DOC)

Table S5 Association of spleen condition with parasitic infections across all host stages (cross-sectional study). Spleen condition was represented in LMMs by spleen weight (the response) adjusted for covariates SVL and its quadratic term, initially averaging slopes for these across life history stages. Models considered all stages sampled and were of the form: Log_10_ spleen weight = LH+Process group+SVL+SVL^2^+LH.SVL+LH.SVL^2^+Parasite variable (random term = Year×Sampling Point×Site). Both of the macroparasite principal component variables (PC^M^ and PC^M main^) and several of the individual macroparasite variables showed significant (or marginally nonsignificant) positive associations with spleen condition. The only significant negative association for a macroparasite was for Listrophoridae (fur mites), which may possibly have been due to an association between poor condition and compromised grooming. The relative development of the spleen is a complex indicator of individual condition, potentially reflecting a combination of generalised individual condition, standing investment in immune defences, and ongoing host responses. The strong positive association of *B. microti* with spleen condition (but not with body or liver condition) is likely to be due to a host response, given that splenomegaly often develops in *Babesia* infections (S*21, 22*). Furthermore, the strong negative association of *Bartonella* spp. with spleen condition (and, again, not with body or liver condition) is likely due to confounding from *B. microti*. *Bartonella* in our data and in previous studies shows a strong negative interaction with *B. microti* (and the significant result for *Bartonella* spp. disappears if *B. microti* is added as an explanatory term to the LMM). Significant positive association in the main hypothesis test is highlighted in orange; significant (or marginally nonsignificant) associations in *post hoc* tests are highlighted in yellow (positive associations) or grey (negative associations).
(DOC)

Table S6 Association of testis condition with parasitic infections in adult males (cross-sectional study). Testis condition was represented in LMMs by testis weight (the response) adjusted for covariates SVL and its quadratic term. Models considered only mature males (testes are minimally developed in immature males) and were of the form: Log_10_ testis weight = Process group+SVL+SVL^2^+Parasite variable (random term = Year×Sampling Point×Site). Both of the macroparasite principal component variables (PC^M^ and PC^M main^) and several individual macroparasite variables showed significant (or marginally nonsignificant) positive associations with testis condition. The only significant negative association was for Listrophoridae (fur mites), which may possibly be due to an association between poor condition and compromised grooming. Significant positive association in the main hypothesis test is highlighted in orange; significant (or marginally nonsignificant) associations in *post hoc* tests are highlighted in yellow (positive associations) or grey (negative associations).
(DOC)

Table S7 Association between body condition and grouped macroparasite infection in different life history stages (cross-sectional study): differing epidemiological reaction norms (ERNs). To assess the heterogeneity of adjusted body condition slopes on grouped macroparasite infection (PC^M main^) across life history stages (i.e., stage-specific ERNs), all life history stages were initially analysed in a single LMM of the form described in Table S3. This included PC^M main^ as the parasite variable and, additionally, a Life History Stage (LH)×PC^M main^ interaction. This interaction was highly significant (*F*_4, 548.9_ = 4.04, *p* = .003), indicating divergent reaction norms. When females were excluded from the analysis, due to the complex and sometimes ambiguous transitions between different reproductive states, there was still highly significant heterogeneity of slopes between mating and nonmating males (*F*_1,316.3_ = 8.98, *p* = .003) (see main text, Figure 1B). The table below presents the slopes (ERNs) of adjusted body condition on PC^M main^ for the life history stages analysed separately. As in Table S3, body condition was represented in LMMs by body weight (the response) adjusted for covariates SVL and its quadratic term. Each life history stage was analyzed separately in models of the form: Body weight = Process group+SVL+SVL^2^+PC^M main^ (random term = Year×Sampling Point×Site). Significant positive associations are highlighted.
(DOC)

Table S8 Association between body condition and immunological gene expression in adult males (cross-sectional study). Body condition was represented in LMMs by body weight (the response) adjusted for covariates SVL and its quadratic term. Body condition was also adjusted for the association with macroparasites (see Table S7) by the inclusion of PC^M main^ as a further covariate. Models included adult males only and were of the form: Body weight = Process group+SVL+SVL^2^+PC^M main^+Immunological variable (random term = Year×Sampling Point×Site). The table shows the results of the main hypothesis tests (H_0_ = condition is unrelated to immunological gene expression) that were initially carried out on the eight immunological principal component (PC) variables, followed by *post hoc* testing of individual variables. PC variables that would be significant at *p*<.01 after multiplicity adjustment (sequential Bonferonni) are highlighted in orange. Individual variables from *post hoc* testing that would be significant at *p*<.01 after a multiplicity adjustment are also highlighted in orange. PC and individual variables that were only significant (or marginally nonsignificant) without multiplicity adjustment are highlighted in yellow (nonsignificant results for *post hoc* testing not shown). PC2^mit-stim^ (which featured a large loading for Gata3^mit-stim^) and Gata3^mit-stim^ itself were the only two significant variables following multiplicity adjustment (for the whole table of tests carried out). Gata3^mit-index^ was also relatively highly significant at an individual level. When Gata3^mit-stim^ was considered in a model of the above form additionally including immature males and different slopes on Gata3^mit-stim^ and PC^M main^ for mature and immature males, there were very significantly different stage-specific Gata3^mit-stim^ slopes, with mature males showing a positive slope and immature males no association (see main text, Figure 1D) (Stage×Gata3^mit-stim^ interaction, *F*_1,211.5_ = 9.96, *p* = .002).
(DOC)

Table S9 Associations between organ condition and mitogen-stimulated Gata3 expression (Gata3^mit-stim^) in adult males (cross-sectional study). Organ condition was represented in LMMs by organ weight (the response) adjusted for covariates SVL and its quadratic term. Organ condition was also adjusted for the association with macroparasites (see Tables S4, S5, S6) by the inclusion of PC^M main^ as a further covariate. Models included adult males only and were of the form: Organ weight = Process group+SVL+SVL^2^+PC^M main^+Log_10_ Gata3^mit-stim^ (random term = Year×Sampling Point×Site). Significant positive associations are highlighted in yellow and significant negative associations in grey.
(DOC)

Table S10 Gata3 expression (Gata3^mit-stim^), overt TB lesions, and body condition (cross-sectional study). *Post hoc* analyses of body condition focussing on TB found a highly significant interaction between overt TB lesions and Gata3 expression in mitogen-stimulated splencocytes (Gata3^mit-stim^) across all stages. This analysis is based on the following LMM: Body weight = Log_10_ Gata3^mit-stim^+LH+Process group+SVL+SVL^2^+LH.SVL+LH.SVL^2^+Overt TB+Overt TB×Log_10_Gata3^mit-stim^ (random term = Year×Sampling Point×Site). There was only a marginally significant overt TB×Gata3^mit-stim^ interaction when the analysis was restricted to adult males (*p* = .08).
(DOC)

Table S11 Associations between parasitic infections and mitogen-stimulated Gata3 expression (Gata3^mit-stim^) in adult males (cross-sectional study). The association of Gata3^mit-stim^ with grouped parasitic infection was analyzed in LMMs of the form: Parasite variable = Process group+SVL+Log_10_ Gata3^mit-stim^ (random term = Year×Sampling Point×Site). In mature males there were significant positive associations with both of the macroparasite principal component variables (PC^M main^ and PC^M^) (see table). When immature males were added to the above model for PC^M main^, with different age-specific slopes, there was significant variation in the Gata3^mit-stim^ slopes, with a positive slope for mature males and a negative slope for immature males (see main text, Figure 1C) (Stage×Gata3^mit-index^ interaction, *F*_1,204.2_ = 16.3, *p* = 7.6×10^−5^). A similar interaction also occurred in the case of PC^M^ (*p* = .031). Significant positive association in main hypothesis test is highlighted in orange; significant association in *post hoc* test is highlighted in yellow.
(DOC)

Table S12 Instantaneous and time-lagged associations between exposure to blood-feeding ectoparasites and Gata3 expression (Gata3^blood^) in peripheral blood in adult males (longitudinal study). (A) Time lagged associations. Association of Gata3^blood^ with infection variables earlier in time (t-1 = 1 mo earlier). When analyzed alone, ticks and small fleas were represented by continuous log-transformed abundance variables. When ticks and small fleas were analyzed together, this was as a binary presence/absence factor (0, no infection; 1, infection with ticks and or fleas). Table shows significant explanatory terms from models of the form: Gata3^blood^ = Weight+Parasite variable (random terms: Year×Sampling point×Site+Assay plate+Individual ID). Results for weight are for the model with a factor for ticks and or small fleas. Significant positive associations highlighted in yellow and significant negative associations in grey. Earlier infection tended to be positively associated with Gata3^blood^ expression. (B) Instantaneous association. Association of Gata3^blood^ with infection at the same time point. When analyzed alone, ticks and small fleas were represented by continuous log-transformed abundance variables. When ticks and small fleas were analyzed together this was as a binary presence/absence factor (0, no infection; 1, infection with ticks and or fleas). Table shows significant explanatory terms from models of the form: Log_10_ Gata3^blood^ = Weight+Parasite variable (random terms: Year×Sampling Point×Site+Assay plate+Individual ID). Results for weight are for the model with a factor for ticks and or small fleas. Significant (or marginally nonsignificant) negative associations highlighted in grey. Contemporaneous infection tended to be negatively associated with Gata3^blood^ expression, perhaps due to some protective (resistance) effect of this response.
(DOC)

Table S13 Time-lagged association between month-on-month weight gain and Gata3 expression (Gata3^blood^) in peripheral blood in adult males (longitudinal study). Association of month-on-month weight gain (adjusted for starting weight) with Gata3^blood^ expression 1 mo earlier. Table shows significant explanatory terms from LMMs of the form: Final weight = Starting weight+Gata3^blood^ (random terms: Year×Sampling Point×Site+Individual ID). Gata3^blood^ was positively associated with adjusted weight gain in the following month. There was no association between Gata3^blood^ and weight gain in the preceding month (analyzed in an equivalent model). The effects of individual parasite variables were examined in these base models (taking each variable in turn), but were nonsignificant. Macroparasite variables examined included the abundance of fleas, ticks, laelapid mites, listrophorid mites, and lice and also an overall ectoparasite index; microparasite variables (presence/absence) included *B. microti*, *Bartonella* spp., and overt TB. Ectoparasite index was an additive score based on the sum of standardized abundances for the different ectoparasites. Significant positive associations are highlighted in yellow.
(DOC)

Table S14 SEM of key variables from cross-sectional study. In the SEM analysis of the cross-sectional data, we searched for models with: the causality indicated by the longitudinal analyses in Tables S12 and S13; higher numbers of exclusively significant (*p*<.05) or near-significant (.05<*p*<.08) parameters; and optimal model fit (root mean square error of approximation, RMSEA) and AIC statistics. The table shows the two best supported models with six or seven significant (or near-significant) parameters. Parameter estimates for model 2 are shown in Table S15. †Pattern of dependency assumed in each model. Gr, overall growth rate (SVL adjusted for age estimated by lens weight); P, infection with key influential macroparasites (PC^M main^, see Table S1); G, Log_10_ Gata3 expression in mitogen-stimulated splenocytes; C, body condition; T, testis condition. Prior to the SEM analysis, all of the variables were adjusted, in general linear models, for spatiotemporal sampling point (sampling time and site nested within year) and for host linear dimensions (SVL+SVL^2^) if SVL did not contribute to the variable already.
(DOC)

Table S15 Parameter estimates for SEM best supported by independent temporal analyses of longitudinal data. Gr, overall growth rate (SVL adjusted for age); P, infection with key influential macroparasites (PC^M main^, see Table S1); G, Log_10_ Gata3 expression in mitogen-stimulated splenocytes; C, body condition; T, testis condition. Prior to the SEM analysis, all of the variables were adjusted, in general linear models, for spatiotemporal sampling point (sampling time and site nested within year) and for host linear dimensions (SVL+SVL^2^) if SVL did not contribute to the variable already. A causal effect of macroparasites (P) upon Gata3 expression (G) and of G upon body condition (C) was independently suggested by analyses of the longitudinal dataset (see respectively, Tables S12 and S13). The stimulation of mammalian Th2 responses (including Gata3 expression) is also a well-known effect of macroparasite infection in experimental settings (see main article).
(DOC)

Table S16 Gata3 expression in peripheral blood (Gata3^blood^) and age-specific primary session return rate (longitudinal study). Return rate of mature males (at the next primary trapping session) (A) was initially analyzed with respect to Gata3 expression in peripheral blood (Gata3^blood^) using a GLMM with binomial errors (trial size = 1). The base model was of the form: Return∼Weight+Gata3^blood^+Peripheral row (random term = Year×Sampling Point×Site+Individual ID). The peripheral row factor accounted for animals captured in the outer trap row of the grid, which might putatively have lower recapture probabilities (as their ranges are more likely to have been centred outside the grid). Other models (not shown) examined the effects of individual parasite variables in this base model (taking each in turn). Macroparasite variables included the abundance of fleas, ticks, laelapid mites, listrophorid mites, and lice and also an overall ectoparasite index; microparasite variables (presence/absence) included *B. microti*, *Bartonella* spp., and overt TB. Ectoparasite index was an additive score based on the sum of standardized abundances for the different ectoparasites. These were only included in the final model if significant. A similar set of models was then investigated (*post hoc*) for all stages (B), additionally including life history stage as a factor. The main effect for Gata3^blood^ was not significant in adult males but was significantly negative in the analysis across all stages (*p* = .003, *F*_1, 863.7_ = 9.02, parameter −0.5852±0.1949). Significant Weight×Gata3^blood^ interactions occurred in both analyses (with Gata3^blood^ having an increasingly positive effect on survival as hosts became heavier). Although return rate might act as a reasonable surrogate for survival (given the high recapture rates observed (see Table S17)), further analyses explicitly modelled survival in the context of variable recapture probability (Table S17). Significant positive associations in tables A–B are highlighted in yellow and significant negative associations in grey. For graphical purposes (Figure 3, main article), predictions of Gata3^blood^ expression in surviving and nonsurviving animals were generated from an LMM: Gata3^blood^ = Weight+Return+Weight.Return+peripheral row+life history stage (random term = Year×Sampling point×Site+Individual ID+Assay plate).
(DOC)

Table S17 Gata3 expression and survival: CJS analysis with time-specific individual covariates (longitudinal study). The effect of Gata3 expression in peripheral blood (Gata3^blood^) was analyzed in CJS models in a subset of males with >1 capture and no or few missing values for Gata3^blood^ (*n* = 107). (This tended to bias the analysis towards larger males due to growth occurring in the capture intervals.) Time-specific body weight covariates were highly significant (LRT, χ^2^ = 17.25, DF = 5, *p* = .004) when added to an optimal base model for survival and recapture that included seasonal (monthly) variation in survival (*φ*) and variation across years in recapture probability (*p*). Gata3^blood^ and Gata3^blood^×Body Weight covariates were then added to the base model already containing body weight covariates. The best resulting models contained covariates for Gata3^blood^ and Gata3^blood^×Body Weight only in the last 2 mo (LRT, χ^2^ = 16.49, DF = 4, *p* = .0024) or last month of the season (LRT, χ^2^ = 15.37, DF = 2, *p* = .0005). In these months, survival tended to increase in larger animals expressing a greater amount of Gata3^blood^. The following parameter values were estimated for a model in which overall *p* and *φ* for the first time interval (*φ*_April→May_) were constrained to be 1. This was because *p* was generally estimated to be very high (>0.8–0.9 in models with constant, year-on-year, or seasonal variation in *p*) and because *φ*_April→May_ was limited by the nature of the data, which only included animals with multiple recaptures. Real function parameters for survival were: *φ*_May→June_ = 0.49±0.07, *φ*_June→July_ = 0.76±0.06, *φ*_July→August_ = 0.87±0.05, *φ*_August→September_ = 0.73±0.05, *φ*_September→October_ = 0.55±0.08. The logit link function parameter for the September Gata3^blood^×Body Weight covariate was: β_September_ = 0.3±0.11.
(DOC)

Table S18 Real-time PCR primers used to measure the expression of immunological genes in peripheral blood.
(DOC)

Table S19 Infection statistics for parasites at Kielder broken down by Locality×Year of Study. A, abundance±standard error. P, prevalence, followed by 95% CI. *Abundance data for Listrophoridae relate to a semiquantitative abundance index. †PCR diagnosis of *Bartonella* spp. and *B. microti* may have had unequal sensitivities in 2008 and 2009 due to the use of different DNA extraction methods. Prevalence data are thus not directly comparable across years.
(DOC)

Methods S1 Supplementary Methods.
(DOC)

## Abbreviations

CJS: Cormack-Jolly-Seber
GLMM: generlalised linear mixed model
LMM: linear mixed model
PCA: principal components analysis
SEM: structural equations modelling
SVL: snout-vent length
TB: tuberculosis
Th2: T-helper type 2
TLR: Toll-like receptor
