# Supplementary figures and images for: An Immunological Marker of Tolerance to Infection in Wild Rodents
Source: PLoS Biol. 2014 Jul 8;12(7):e1001901. doi: 10.1371/journal.pbio.1001901 (PMC4086718; doi:10.1371/journal.pbio.1001901)

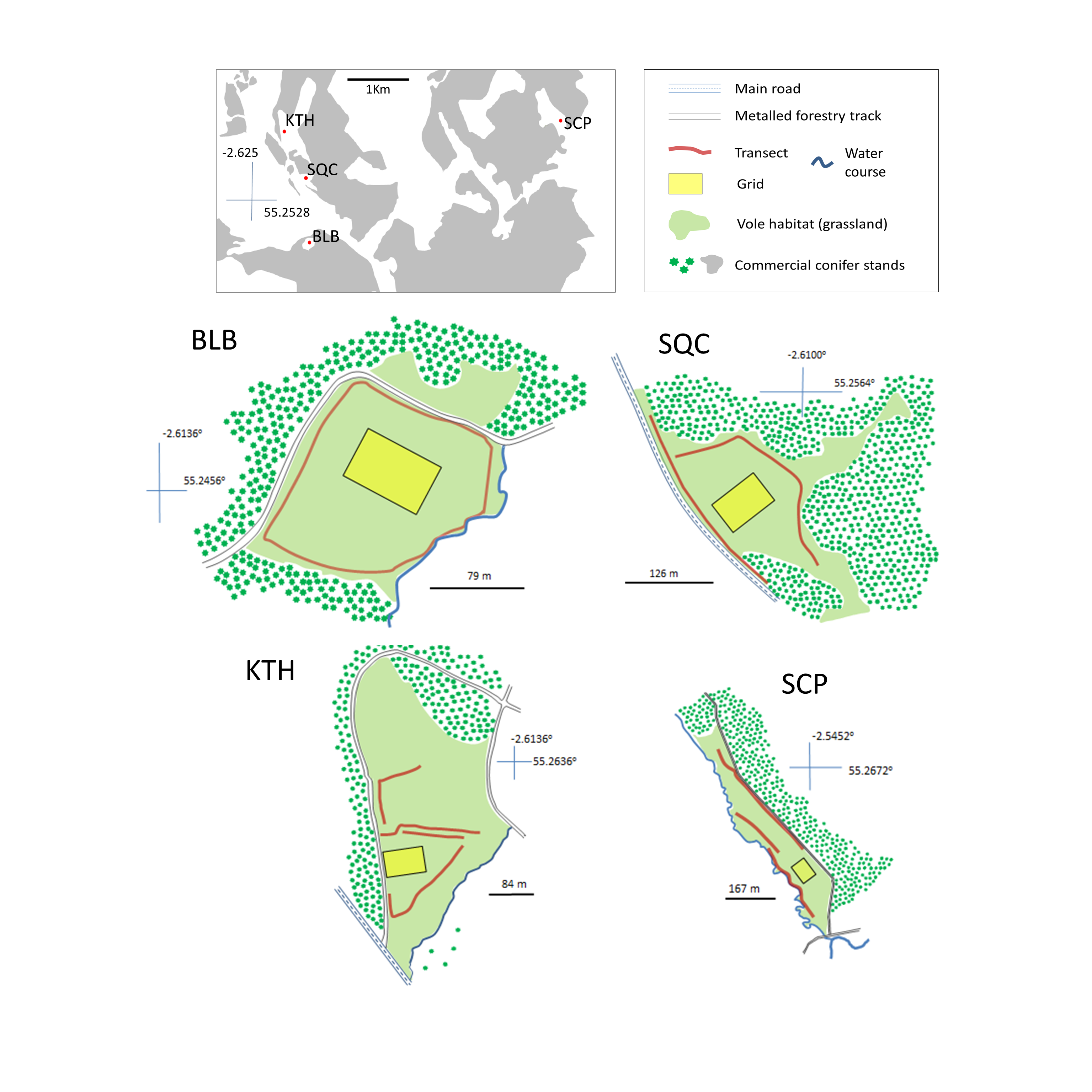

Supplement: Figure S1 — Study sites. (TIF) [file pbio.1001901.s001.tif]

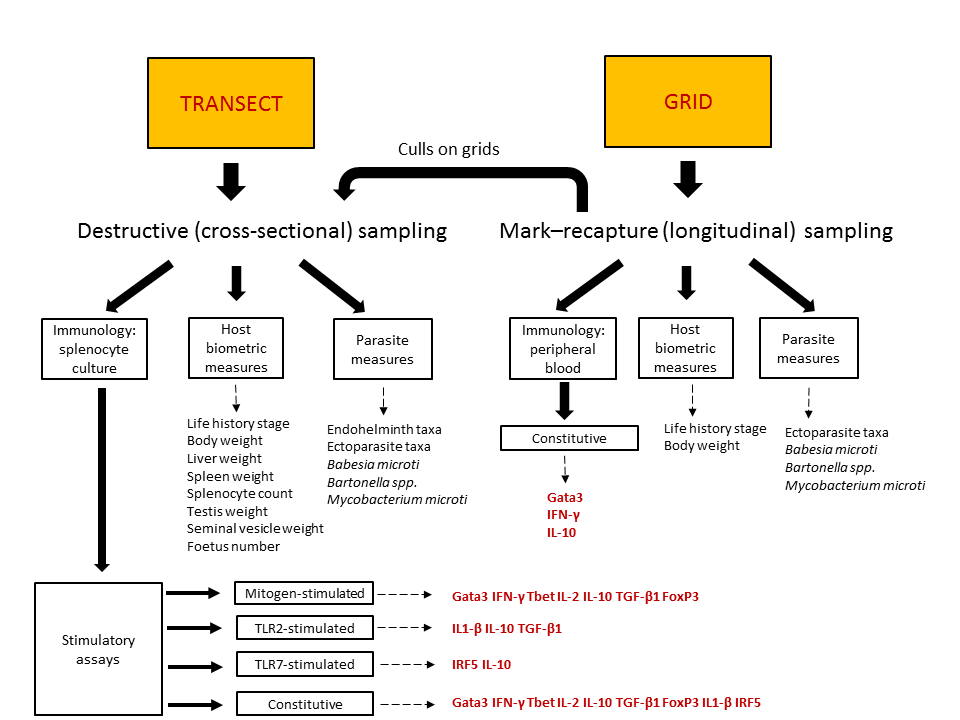

Supplement: Figure S2 — Sampling and measurements. Design replicated on two sites in 2008–2009 and two different sites in 2009–2010. Processing steps indicated by solid arrows and measurements by dashed arrows. Gene expression measurements shown in red. For timeline, see Figure S3. (TIF) [file pbio.1001901.s002.tif]

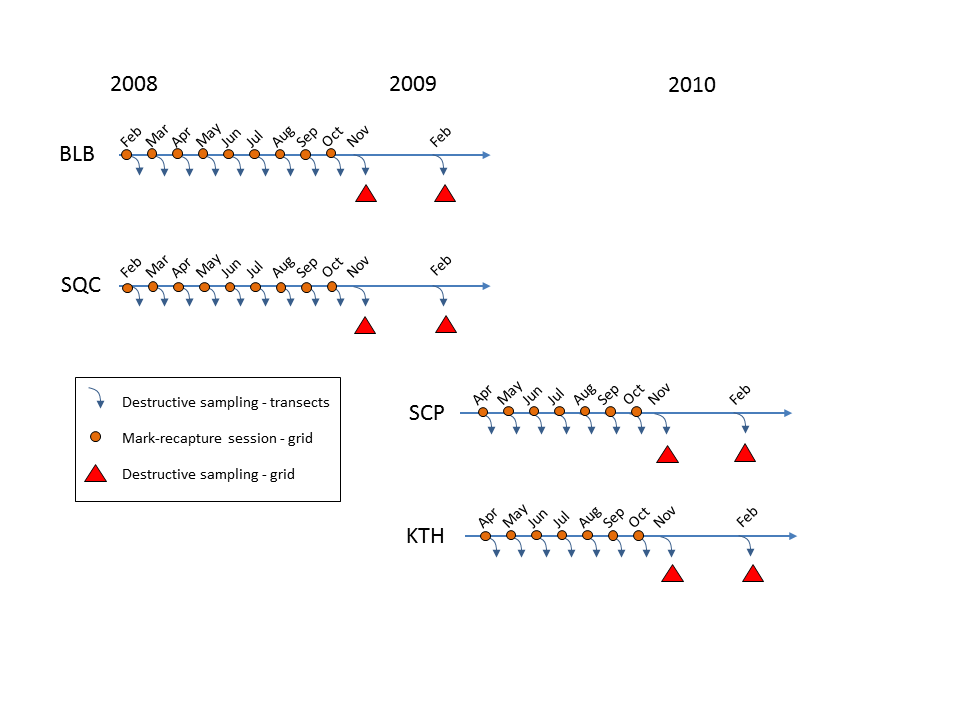

Supplement: Figure S3 — Timeline of sampling. (TIF) [file pbio.1001901.s003.tif]

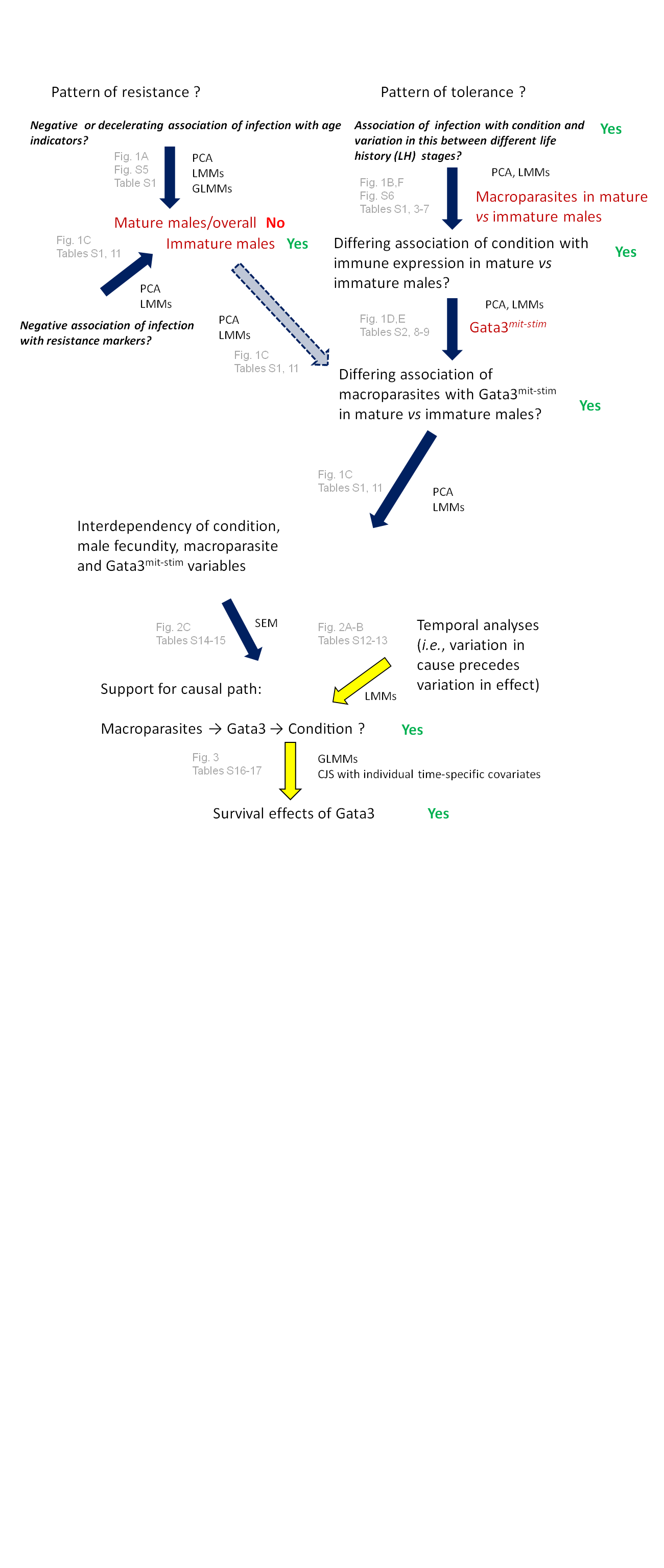

Supplement: Figure S4 — Flow of main analysis. In order to avoid problems of interpretation due to multiplicity of tests, we carried out our analyses in a stepwise fashion, with each continuation step conditional on significant results in a single or small number of main hypothesis tests in the previous step. For parasite and immune expression data, these main tests initially used reduced variables (principal component, PC, scores). Significant main tests involving PC variables were followed by exhaustive post hoc testing of individual variables. Arrows indicate steps in the analysis associated with a particular hypothesis test or tests. Blue arrows indicate steps using data from the cross-sectional study (from which a wider range of more precise immunological, biometric, and parasitological measurements was available). Where a particular individual variable or stratum of hosts was strongly implicated at a given step of the analysis and is focussed upon in the subsequent analysis, this is indicated in red to the right. Yellow arrows indicate steps using longitudinal data (where temporal sequences allowed stronger inference on cause and effect and the estimation of survival). Details of the respective analysis steps are provided in the figures and tables indicated on the left of each arrow (in grey) and the analytical methods employed are indicated on the right (in black) (PCA, principal components analysis; LMM, linear mixed model; GLMM, generalized linear mixed model; SEM, structural equations modelling; CJS, Cormack-Jolly-Seber survival analysis). (TIF) [file pbio.1001901.s004.tif]

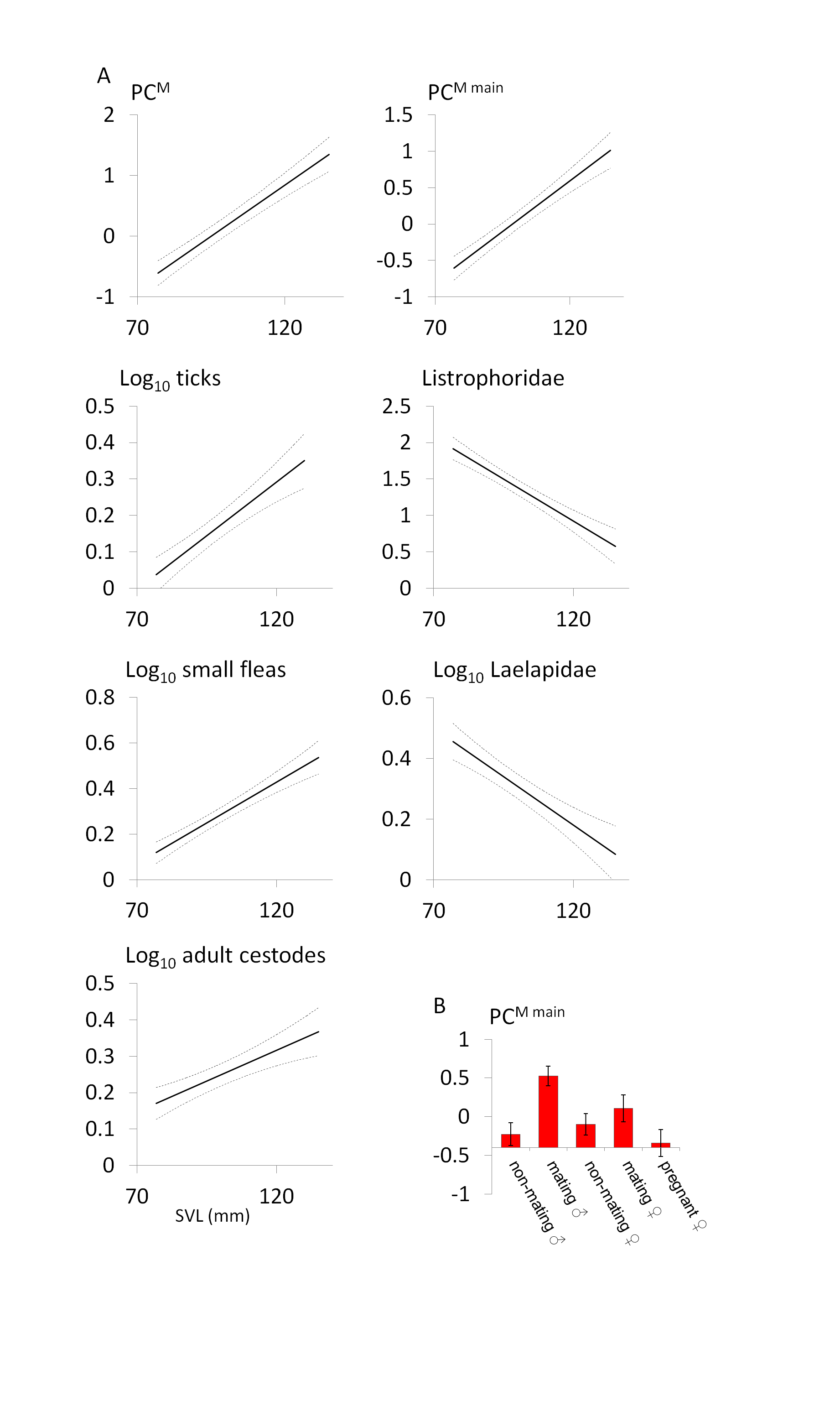

Supplement: Figure S5 — Overall associations between macroparasite abundance and host age indicators and life history stage. (A) Associations between parasite abundance and host size and age indicators, averaging slopes across life history groups. Plotted relationships are predictions (±1 standard error) from LMMs of the form: Parasite variable = Process day+LH+SVL (random component: Year×Sampling Point×Site). A quadratic term for SVL was initially included in models, and equivalent models were also analysed with lens weight terms replacing the SVL terms (with similar results). There were highly significant positive linear associations with the principal component representing key macroparasite species, PCM main (see Table S1), and with the individual key macroparasite taxa (small fleas, ticks, and adult cestodes). There were no significant decelerating quadratic terms for SVL or lens weight. The key taxa were common species (>20% prevalence) in strong contact with the host immune system due to feeding habit or definitive site (see Table S1). The common detritivorous ectoparasitic mites (not expected to be in strong contact with the host immune system and perhaps better considered commensals rather than parasites) were significantly negatively linearly associated with SVL and lens weight. This might be related to increased grooming efficiency in older animals or to nidicolous transmission foci. PCM, the principal component reflecting variation in the abundance of all macroparasites, strongly contrasted the abundances of key influential species (with positive loadings) with the abundances of common detritivorous mites (with negative loadings) and was thus also positively linearly associated with SVL and lens weight. There was thus no evidence of ongoing buildups of acquired resistance to the key macroparasitic throughout the lives of individuals, although analyses stratified by life history stage, with different macroparasite on age indicator slopes, indicated that resistance might build up [file pbio.1001901.s005.tif]
