# Supplementary material for: An Immunological Marker of Tolerance to Infection in Wild Rodents
Source: PLoS Biol. 2014 Jul 8;12(7):e1001901. doi: 10.1371/journal.pbio.1001901 (PMC4086718; doi:10.1371/journal.pbio.1001901)
Supplement: Table S1 — Multivariate reduction of macroparasite data (cross-sectional study). PCA was used to generate single composite variables (first principal components, PC1) representing the main axis of covariation amongst sets of macroparasite variables. Scores from these PC1 were then used in subsequent analyses to represent “grouped” macroparasite variation. An initial PCA included all of the macroparasite variables. PC1 (designated PCM) was dominated by larger loadings for the more common species. These loadings strongly contrasted laelapid and listrophorid mite abundances with those of small fleas, ticks, and intestinal cestodes. A second PCA was carried out including only common macroparasite species that feed directly on host tissues (small fleas and ticks) or that have an intimate association with host mucosal surfaces and adsorb soluble nutrients utilizable by the host (adult cestodes). This excluded all rare species (prevalence <20%). It also excluded common species that do not feed directly on host tissues, do not have an intimate association with host mucosal surfaces, and do not adsorb soluble nutrients. This was the case for listrophorid and laelapid mites, which are primarily deritivores, and pinworms (Syphacia), which occur free in the lumen of the colon and feed on bacteria. (Some of these species, especially the detritivorous mites, might better be considered as commensals rather than genuine parasites under normal conditions.) This second PCA produced a very dominant first component (PCM main) with large loadings of similar magnitude and the same sign for small fleas, ticks, and adult cestodes, indicating a pattern of general positive covariation among these. In subsequent analyses, PCM main was used as the primary index of macroparasite infection pressure (due to its strong representation of the species likely to be most influential), but PCM was also analyzed secondarily (with similar results). (DOC) [file pbio.1001901.s006.doc]

|  | **PC^M^** | **PC^M main^** |
| --- | --- | --- |
|  |  |  |
| **% variation explained** | 17% | 47% |
| **Eigenvalue** | 2.16 | 1.41 |
| **Variable loadings:** |  | |
| Log_10_ Total fleas | **0.37** | **0.57** |
| Log_10_ Mole fleas | 0.04 |  |
| Log_10_ Lice | 0.19 |  |
| Log_10_ Total ticks | **0.38** | **0.56** |
| Log_10_ Myobiidae | 0.22 |  |
| Log_10_ Laelapidae | **-0.38** |  |
| Listrophoridae | **-0.46** |  |
| Log_10_ Ear mites | 0.03 |  |
| Log_10_ *S. nigeriana* | 0.25 |  |
| Log_10_ *T. arvicolae* | 0.03 |  |
| Log_10_ *H. laevis* | 0.17 |  |
| Log_10_ Total adult cestodes | **0.42** | **0.60** |
| Log_10_ Total larval cestodes | 0.10 |  |
