# Supplementary material for: An Immunological Marker of Tolerance to Infection in Wild Rodents
Source: PLoS Biol. 2014 Jul 8;12(7):e1001901. doi: 10.1371/journal.pbio.1001901 (PMC4086718; doi:10.1371/journal.pbio.1001901)
Supplement: Table S2 — Multivariate reduction of immune expression data (cross-sectional study). PCA was used to reduce sets of immune expression variables into smaller sets of nonredundant composite variables (principal components, PC) reflecting major axes of covariation among the starting variables. Scores from these PCs were then used in subsequent analyses to represent grouped immune responses (i.e., reflecting the major patterns of covariation). Immune expression data were considered in two different ways: (1) expression under stimulatory conditions relative to a calibrator sample (RQ values) and (2) expression indexed to a corresponding unstimulated control culture. Each of these sets of variables was reduced separately, with distinct analyses being carried out within each set for data from TLR-stimulated and mitogen-stimulated cultures. This gave four separate PCAs, from each of which we considered the two largest components (PC1 and PC2) in further analyses. The components were designated: PC1tlr-stim and PC2tlr-stim (respectively, PC1 and PC2 for nonindexed relative expression data from TLR-stimulated cultures); PC1tlr-index and PC2tlr-index (respectively, PC1 and PC2 for indexed expression data from TLR-stimulated cultures); PC1mit-stim and PC2mit-stim (respectively, PC1 and PC2 for nonindexed relative expression data from mitogen-stimulated cultures); and PC1mit-index and PC2mit-index (respectively, PC1 and PC2 for indexed expression data from mitogen-stimulated cultures). (DOC) [file pbio.1001901.s007.doc]

|  | **PC1**  **^tlr-stim^** | **PC2**  **^tlr-stim^** | **PC1**  **^tlr-index^** | **PC2**  **^tlr-index^** | **PC1**  **^mit-stim^** | **PC2**  **^mit-stim^** | **PC1**  **^mit-index^** | **PC2**  **^mit-index^** |
| --- | --- | --- | --- | --- | --- | --- | --- | --- |
|  |  |  |  |  |  |  |  |  |
| **% variation explained** | 32.4 | 20.8 | 44.3 | 18.7 | 30.8 | 21.0 | 35.9 | 19.6 |
| **Eigenvalue** | 1.62 | 1.04 | 2.21 | 0.93 | 2.16 | 1.47 | 2.51 | 1.37 |
|  |  | |  |  |  |  |  |  |
| **Variable loadings:** |  | |  |  |  |  |  |  |
| Log_10_ IL-1β **^tlr2-stim^** | **0.50** | **-0.49** |  |  |  |  |  |  |
| Log_10_ IL-10 **^tlr2-stim^** | **0.51** | 0.08 |  |  |  |  |  |  |
| Log_10_ TGF-β1 **^tlr2-stim^** | **0.43** | **-0.42** |  |  |  |  |  |  |
| Log_10_ IRF5 **^tlr7-stim^** | **0.31** | **0.64** |  |  |  |  |  |  |
| Log_10_ IL-10 **^tlr7-stim^** | **0.46** | **0.41** |  |  |  |  |  |  |
| Log_10_ IL-1β **^tlr2-index^** |  |  | **0.43** | -**0.32** |  |  |  |  |
| Log_10_ IL-10 **^tlr2-index^** |  |  | **0.53** | 0.06 |  |  |  |  |
| Log_10_ TGF-β1 **^tlr2-index^** |  |  | **0.35** | **-0.68** |  |  |  |  |
| Log_10_ IRF5 **^tlr7-index^** |  |  | **0.55** | 0.25 |  |  |  |  |
| Log_10_ IL-10 **^tlr7-index^** |  |  | **0.33** | **0.61** |  |  |  |  |
| Log_10_ IFN-γ **^mit-stim^** |  |  |  |  | **0.49** | 0.06 |  |  |
| Log_10_ Tbet **^mit-stim^** |  |  |  |  | **0.55** | -0.10 |  |  |
| Log_10_ IL-2 **^mit-stim^** |  |  |  |  | **0.34** | **0.42** |  |  |
| Log_10_ Gata3**^mit-stim^** |  |  |  |  | 0.01 | **0.56** |  |  |
| Log_10_ IL-10 **^mit-stim^** |  |  |  |  | **0.31** | 0.22 |  |  |
| Log_10_ TGF-β1**^mit-stim^** |  |  |  |  | **0.48** | -0.20 |  |  |
| Log_10_ FoxP3**^mit-stim^** |  |  |  |  | 0.15 | **-0.64** |  |  |
| Log_10_ IFN-γ **^mit-index^** |  |  |  |  |  |  | **0.40** | **0.46** |
| Log_10_ Tbet **^mit-index^** |  |  |  |  |  |  | **0.37** | **0.54** |
| Log_10_ IL-2 **^mit-index^** |  |  |  |  |  |  | **0.45** | -0.26 |
| Log_10_ Gata3**^mit-index^** |  |  |  |  |  |  | 0.24 | -0.20 |
| Log_10_ IL-10 **^mit-index^** |  |  |  |  |  |  | **0.31** | 0.27 |
| Log_10_ TGF-β1**^mit-index^** |  |  |  |  |  |  | **0.48** | -0.27 |
| Log_10_ FoxP3**^mit-index^** |  |  |  |  |  |  | **0.34** | **-0.50** |
