# Supplementary material for: An Immunological Marker of Tolerance to Infection in Wild Rodents
Source: PLoS Biol. 2014 Jul 8;12(7):e1001901. doi: 10.1371/journal.pbio.1001901 (PMC4086718; doi:10.1371/journal.pbio.1001901)
Supplement: Table S3 — Association of body condition with parasitic infections across all host stages (cross-sectional study). Body condition was represented in LMMs by body weight (the response) adjusted for covariates SVL and its quadratic term, initially averaging slopes for these across life history stages. Models considered all of the sampled stages and were of the form: Body weight = LH+Process group+SVL+SVL2+LH.SVL+LH.SVL2+Parasite variable (random term = Year×Sampling Point×Site). Both of the macroparasite principal component variables (PCM and PCM main), several of the individual macroparasite variables, and presence/absence of overt TB all showed significant positive associations with body condition. The only negative association was for Listrophoridae (fur mites), which may possibly have been due to an association between poor condition and compromised grooming. Significant positive association in the main hypothesis test is highlighted in orange; significant associations in post hoc tests are highlighted in yellow (positive associations) or grey (negative associations). (DOC) [file pbio.1001901.s008.doc]

| **Term** | **Test statistic** | ***P*** | **Parameter ± standard error** |
| --- | --- | --- | --- |
| **PC^M^** | ***F*_1, 463.3_ = 26.58** | **<5 × 10^-7^** | **0.6877 ± 0.1334** |
| **PC^M main^** | ***F*_1, 542.1_ = 27.90** | **<5 × 10^-7^** | **0.7766 ± 0.1483** |
| **Log_10_ Total fleas** | ***F*_1, 556.0_ = 5.62** | **0.018** | **1.173 ± 0.4945** |
| Log_10_ Mole fleas | *F*_1, 534.7_ = 0.50 | 0.479 |  |
| **Log_10_ Lice** | ***F*_1, 526.4_ = 6.96** | **0.009** | **2.234 ± 0.4782** |
| **Log_10_ Total ticks** | ***F*_1, 550.9_ = 29.04** | **<5 × 10^-7^** | **2.577 ± 0.8465** |
| Log_10_ Myobiidae | *F*_1, 535.8_= 0.61 | 0.434 |  |
| Log_10_ Laelapidae | *F*_1, 555.9_= 0.01 | 0.925 |  |
| **Listrophoridae** | ***F*_1, 556.0_= 6.10** | **0.014** | **-0.3688 ± 0.1493** |
| Log_10_ Ear mites | *F*_1, 515.6_= 0.45 | 0.504 |  |
| Log_10_ *S. nigeriana* | *F*_1, 538.4_= 1.38 | 0.241 |  |
| Log_10_ *T. arvicolae* | *F*_1, 552.2_= 2.10 | 0.148 |  |
| Log_10_ *H. laevis* | *F*_1, 531.5_= 0.10 | 0.747 |  |
| **Log_10_ Total adult cestodes** | ***F*_1, 552.0_= 4.19** | **0.041** | **1.107 ± 0.5407** |
| **Log_10_ Total larval cestodes** | ***F*_1, 529.0_= 4.22** | **0.040** | **5.889 ± 2.8654** |
|  | | | |
| *Bartonella* spp. | *F*_1, 470.7_= 2.54 | 0.112 |  |
| *B. microti* | *F*_1, 445.4_= 1.31 | 0.253 |  |
| **TB with overt lesion** | ***F*_1, 521.4_= 5.66** | **0.018** | **Lesion^+^ 1.9602 ± 0.8239** |
