# Supplementary material for: An Immunological Marker of Tolerance to Infection in Wild Rodents
Source: PLoS Biol. 2014 Jul 8;12(7):e1001901. doi: 10.1371/journal.pbio.1001901 (PMC4086718; doi:10.1371/journal.pbio.1001901)
Supplement: Table S5 — Association of spleen condition with parasitic infections across all host stages (cross-sectional study). Spleen condition was represented in LMMs by spleen weight (the response) adjusted for covariates SVL and its quadratic term, initially averaging slopes for these across life history stages. Models considered all stages sampled and were of the form: Log10 spleen weight = LH+Process group+SVL+SVL2+LH.SVL+LH.SVL2+Parasite variable (random term = Year×Sampling Point×Site). Both of the macroparasite principal component variables (PCM and PCM main) and several of the individual macroparasite variables showed significant (or marginally nonsignificant) positive associations with spleen condition. The only significant negative association for a macroparasite was for Listrophoridae (fur mites), which may possibly have been due to an association between poor condition and compromised grooming. The relative development of the spleen is a complex indicator of individual condition, potentially reflecting a combination of generalised individual condition, standing investment in immune defences, and ongoing host responses. The strong positive association of B. microti with spleen condition (but not with body or liver condition) is likely to be due to a host response, given that splenomegaly often develops in Babesia infections (S21, 22). Furthermore, the strong negative association of Bartonella spp. with spleen condition (and, again, not with body or liver condition) is likely due to confounding from B. microti. Bartonella in our data and in previous studies shows a strong negative interaction with B. microti (and the significant result for Bartonella spp. disappears if B. microti is added as an explanatory term to the LMM). Significant positive association in the main hypothesis test is highlighted in orange; significant (or marginally nonsignificant) associations in post hoc tests are highlighted in yellow (positive associations) or grey (negative associations). (DOC) [file pbio.1001901.s010.doc]

| **Term** | **Test statistic** | ***P*** | **Parameter ± standard error** |
| --- | --- | --- | --- |
| **PC^M^** | ***F*_1, 269.9_ = 17.48** | **3.9 × 10^-5^** | **0.0477 ± 0.0114** |
| **PC^M main^** | ***F*_1, 376.6_ = 18.42** | **2.3 × 10^-5^** | **0.0578 ± 0.0134** |
| **Log_10_ Total fleas** | ***F*_1, 341.5_ = 2.92** | **0.089** | **0.0780 ± 0.0457** |
| Log_10_ Mole fleas | *F*_1, 555.4_ = 0.12 | 0.724 |  |
| Log_10_ Lice | *F*_1, 551.8_ = 2.47 | 0.116 |  |
| **Log_10_ Total ticks** | ***F*_1, 518.6_ = 10.56** | **0.001** | **0.1506 ± 0.0464** |
| Log_10_ Myobiidae | *F*_1, 554.9_ = 0.30 | 0.583 |  |
| Log_10_ Laelapidae | *F*_1, 346.1_ = 0.00 | 0.954 |  |
| **Listrophoridae** | ***F*_1, 448.3_ = 6.09** | **0.014** | **-0.0348 ± 0.0141** |
| Log_10_ Ear mites | *F*_1, 547.1_ = 0.21 | 0.645 |  |
| Log_10_ *S. nigeriana* | *F*_1, 547.2_ = 1.24 | 0.266 |  |
| Log_10_ *T. arvicolae* | *F*_1, 427.8_ = 0.01 | 0.909 |  |
| Log_10_ *H. laevis* | *F*_1, 555.7_ = 0.68 | 0.410 |  |
| **Log_10_ Total adult cestodes** | ***F*_1, 338.9_ = 4.14** | **0.043** | **0.0125 ± 0.0061** |
| Log_10_ Total larval cestodes | *F*_1, 556.9_ = 0.15 | 0.698 |  |
|  | | | |
| ***Bartonella* spp.** | ***F*_1, 437.9_ = 11.64** | **7.1 × 10^-4^** | **-0.1108 ± 0.0325** |
| ***B. microti*** | ***F*_1, 456.2_ = 270.39** | **<5 × 10^-7^** | **0.4658 ± 0.0283** |
| TB with overt lesion | *F*_1, 556.0_ = 0.10 | 0.754 |  |
