# Supplementary material for: An Immunological Marker of Tolerance to Infection in Wild Rodents
Source: PLoS Biol. 2014 Jul 8;12(7):e1001901. doi: 10.1371/journal.pbio.1001901 (PMC4086718; doi:10.1371/journal.pbio.1001901)
Supplement: Table S6 — Association of testis condition with parasitic infections in adult males (cross-sectional study). Testis condition was represented in LMMs by testis weight (the response) adjusted for covariates SVL and its quadratic term. Models considered only mature males (testes are minimally developed in immature males) and were of the form: Log10 testis weight = Process group+SVL+SVL2+Parasite variable (random term = Year×Sampling Point×Site). Both of the macroparasite principal component variables (PCM and PCM main) and several individual macroparasite variables showed significant (or marginally nonsignificant) positive associations with testis condition. The only significant negative association was for Listrophoridae (fur mites), which may possibly be due to an association between poor condition and compromised grooming. Significant positive association in the main hypothesis test is highlighted in orange; significant (or marginally nonsignificant) associations in post hoc tests are highlighted in yellow (positive associations) or grey (negative associations). (DOC) [file pbio.1001901.s011.doc]

| **Term** | **Test statistic** | ***P*** | **Parameter ± standard error** |
| --- | --- | --- | --- |
| **PC^M^** | ***F*_1, 228.6_ = 24.81** | **<5 × 10^-7^** | **0.0421 ± 0.0085** |
| **PC^M main^** | ***F*_1, 212.0_ = 10.10** | **0.002** | **0.0289 ± 0.0091** |
| Log_10_ Total fleas | *F*_1, 212.6_ = 0.82 | 0.366 |  |
| Log_10_ Mole fleas | *F*_1, 200.2_ = 0.22 | 0.642 |  |
| Log_10_ Lice | *F*_1, 194.5_ = 0.48 | 0.487 |  |
| **Log_10_ Total ticks** | ***F*_1, 208.4_ = 6.90** | **0.009** | **0.0714 ± 0.0272** |
| **Log_10_ Myobiidae** | ***F*_1, 193.9_ = 2.89** | **0.091** | **0.0369 ± 0.0217** |
| Log_10_ Laelapidae | *F*_1, 206.7_ = 0.00 | 0.951 |  |
| **Listrophoridae** | ***F*_1, 214.6_ = 10.28** | **0.002** | **-0.0356 ± 0.0111** |
| Log_10_ Ear mites | *F*_1, 195.8_ = 0.93 | 0.335 |  |
| Log_10_ *S. nigeriana* | *F*_1, 193.5_ = 2.12 | 0.147 |  |
| Log_10_ *T. arvicolae* | *F*_1, 202.8_ = 0.51 | 0.476 |  |
| Log_10_ *H. laevis* | *F*_1, 194.5_ = 0.55 | 0.459 |  |
| **Log_10_ Total adult cestodes** | ***F*_1, 205.9_ = 4.66** | **0.032** | **0.0779 ± 0.0361** |
| Log_10_ Total larval cestodes | *F*_1, 194.7_ = 1.88 | 0.171 |  |
|  | | | |
| *Bartonella* spp. | *F*_1, 195.3_ = 0.51 | 0.477 |  |
| *B. microti* | *F*_1, 186.5_ = 0.00 | 0.993 |  |
| TB with overt lesion | *F*_1, 192.2_ = 0.82 | 0.366 |  |
