# Supplementary material for: An Immunological Marker of Tolerance to Infection in Wild Rodents
Source: PLoS Biol. 2014 Jul 8;12(7):e1001901. doi: 10.1371/journal.pbio.1001901 (PMC4086718; doi:10.1371/journal.pbio.1001901)
Supplement: Table S7 — Association between body condition and grouped macroparasite infection in different life history stages (cross-sectional study): differing epidemiological reaction norms (ERNs). To assess the heterogeneity of adjusted body condition slopes on grouped macroparasite infection (PCM main) across life history stages (i.e., stage-specific ERNs), all life history stages were initially analysed in a single LMM of the form described in Table S3. This included PCM main as the parasite variable and, additionally, a Life History Stage (LH)×PCM main interaction. This interaction was highly significant (F 4, 548.9 = 4.04, p = .003), indicating divergent reaction norms. When females were excluded from the analysis, due to the complex and sometimes ambiguous transitions between different reproductive states, there was still highly significant heterogeneity of slopes between mating and nonmating males (F 1,316.3 = 8.98, p = .003) (see main text, Figure 1B). The table below presents the slopes (ERNs) of adjusted body condition on PCM main for the life history stages analysed separately. As in Table S3, body condition was represented in LMMs by body weight (the response) adjusted for covariates SVL and its quadratic term. Each life history stage was analyzed separately in models of the form: Body weight = Process group+SVL+SVL2+PCM main (random term = Year×Sampling Point×Site). Significant positive associations are highlighted. (DOC) [file pbio.1001901.s012.doc]

| **Stage** | **Test statistic for PC^M main^** | ***P*** | **Parameter ± standard error** |
| --- | --- | --- | --- |
| Non-mating males | *F*_1, 101.8_ = 0.28 | 0.600 |  |
| **Mating males** | ***F*_1, 220.0_ = 14.75** | **1.6 × 10^-4^** | **0.8132 ± 0.2117** |
| **Non-mating females** | ***F*_1, 113.7_ = 4.34** | **0.039** | **0.3565 ± 0.1711** |
| **Mating females** | ***F*_1, 41.8_ = 14.48** | **4.6 × 10^-4^** | **1.756 ± 0.461** |
| Pregnant females | *F*_1, 46.4_ = 0.51 | 0.480 |  |
