# Supplementary material for: An Immunological Marker of Tolerance to Infection in Wild Rodents
Source: PLoS Biol. 2014 Jul 8;12(7):e1001901. doi: 10.1371/journal.pbio.1001901 (PMC4086718; doi:10.1371/journal.pbio.1001901)
Supplement: Table S8 — Association between body condition and immunological gene expression in adult males (cross-sectional study). Body condition was represented in LMMs by body weight (the response) adjusted for covariates SVL and its quadratic term. Body condition was also adjusted for the association with macroparasites (see Table S7) by the inclusion of PCM main as a further covariate. Models included adult males only and were of the form: Body weight = Process group+SVL+SVL2+PCM main+Immunological variable (random term = Year×Sampling Point×Site). The table shows the results of the main hypothesis tests (H0 = condition is unrelated to immunological gene expression) that were initially carried out on the eight immunological principal component (PC) variables, followed by post hoc testing of individual variables. PC variables that would be significant at p<.01 after multiplicity adjustment (sequential Bonferonni) are highlighted in orange. Individual variables from post hoc testing that would be significant at p<.01 after a multiplicity adjustment are also highlighted in orange. PC and individual variables that were only significant (or marginally nonsignificant) without multiplicity adjustment are highlighted in yellow (nonsignificant results for post hoc testing not shown). PC2mit-stim (which featured a large loading for Gata3mit-stim) and Gata3mit-stim itself were the only two significant variables following multiplicity adjustment (for the whole table of tests carried out). Gata3mit-index was also relatively highly significant at an individual level. When Gata3mit-stim was considered in a model of the above form additionally including immature males and different slopes on Gata3mit-stim and PCM main for mature and immature males, there were very significantly different stage-specific Gata3mit-stim slopes, with mature males showing a positive slope and immature males no association (see main text, Figure 1D) (Stage×Gata3mit-stim interaction, F 1,211.5 = 9.96, p = .002). (DOC) [file pbio.1001901.s013.doc]

| **Term** | **Test statistic** | ***P*** | **Parameter ± standard error** |
| --- | --- | --- | --- |
| PC1 ^mit-stim^ | *F*_1, 144.5_ = 0.97 | 0.326 |  |
| **PC2 ^mit-stim^** | ***F*_1, 141.7_ = 17.33** | **5.4 × 10^-5^** | **1.024 ± 0.2459** |
| **PC1 ^tlr-stim^** | ***F*_1, 136.9_ = 4.73** | **0.031** | **0.4672 ± 0.2148** |
| PC2 ^tlr-stim^ | *F*_1, 139.3_ = 0.26 | 0.614 |  |
| PC1 ^mit-index^ | *F*_1, 44.5_ = 0.06 | 0.814 |  |
| **PC2 ^mit-index^** | ***F*_1, 46.0_ = 5.78** | **0.020** | **-1.061 ± 0.4415** |
| PC1 ^tlr-index^ | *F*_1, 56.0_ = 1.33 | 0.253 |  |
| PC2 ^tlr-index^ | *F*_1, 47.4_ = 0.39 | 0.533 |  |
| **IL-10 ^tlr7-stim^** | ***F*_1, 165.1_ = 4.66** | **0.032** | **1.149 ± 0.5325** |
| **IL-1β ^tlr2-stim^** | ***F*_1, 165.5_ = 4.54** | **0.035** | **1.717 ± 0.8061** |
| **TGF-β1 ^tlr2-stim^** | ***F*_1, 154.4_ = 3.27** | **0.072** | **2.687 ± 1.4848** |
| **Tbet ^mit-stim^** | ***F*_1, 144.1_ = 3.29** | **0.072** | **-2.675 ± 1.474** |
| **FoxP3 ^mit-stim^** | ***F*_1, 145.6_ = 7.50** | **0.007** | **-3.110 ± 1.136** |
| **IL-2^mit-stim^** | ***F*_1, 146.6_ = 3.79** | **0.053** | **1.722 ± 0.885** |
| **Gata3^mit-stim^** | ***F*_1, 131.6_ = 16.83** | **7.1 × 10^-5^** | **2.934 ± 0.715** |
| **IL-2 ^mit-index^** | ***F*_1, 110.7_ = 5.64** | **0.019** | **1.782 ± 0.750** |
| **Gata3 ^mit-index^** | ***F*_1, 79.9_ = 7.34** | **0.008** | **1.197 ± 0.442** |
