# Supplementary material for: An Immunological Marker of Tolerance to Infection in Wild Rodents
Source: PLoS Biol. 2014 Jul 8;12(7):e1001901. doi: 10.1371/journal.pbio.1001901 (PMC4086718; doi:10.1371/journal.pbio.1001901)
Supplement: Table S9 — Associations between organ condition and mitogen-stimulated Gata3 expression (Gata3mit-stim) in adult males (cross-sectional study). Organ condition was represented in LMMs by organ weight (the response) adjusted for covariates SVL and its quadratic term. Organ condition was also adjusted for the association with macroparasites (see Tables S4, S5, S6) by the inclusion of PCM main as a further covariate. Models included adult males only and were of the form: Organ weight = Process group+SVL+SVL2+PCM main+Log10 Gata3mit-stim (random term = Year×Sampling Point×Site). Significant positive associations are highlighted in yellow and significant negative associations in grey. (DOC) [file pbio.1001901.s014.doc]

| **Stage** | **Test statistic for Gata3^mit-stim^** | P | **Parameter ± standard error** |
| --- | --- | --- | --- |
| **Liver** | ***F*_1, 149.5_ = 5.00** | **0.027** | **0.1670 ± 0.0747** |
| Log_10_ Spleen | *F*_1, 149.0_ = 1.66 | 0.199 |  |
| **Testis** | ***F*_1, 120.8_ = 6.18** | **0.014** | **-0.04737 ± 0.019045** |
