# Supplementary material for: An Immunological Marker of Tolerance to Infection in Wild Rodents
Source: PLoS Biol. 2014 Jul 8;12(7):e1001901. doi: 10.1371/journal.pbio.1001901 (PMC4086718; doi:10.1371/journal.pbio.1001901)
Supplement: Table S10 — Gata3 expression (Gata3mit-stim), overt TB lesions, and body condition (cross-sectional study). Post hoc analyses of body condition focussing on TB found a highly significant interaction between overt TB lesions and Gata3 expression in mitogen-stimulated splencocytes (Gata3mit-stim) across all stages. This analysis is based on the following LMM: Body weight = Log10 Gata3mit-stim+LH+Process group+SVL+SVL2+LH.SVL+LH.SVL2+Overt TB+Overt TB×Log10Gata3mit-stim (random term = Year×Sampling Point×Site). There was only a marginally significant overt TB×Gata3mit-stim interaction when the analysis was restricted to adult males (p = .08). (DOC) [file pbio.1001901.s015.doc]

**-**

| **Stage** | **Test statistic** | P | **Parameter ± standard error** |
| --- | --- | --- | --- |
| Process day | *F*_1, 368.4_ = 0.10 | 0.748 |  |
| LH.SVL | *F*_4, 371.8_ = 5.42 | <0.001 |  |
| LH.SVL^2^ | *F*_4, 371.6_ = 5.35 | <0.001 |  |
| Overt TB.Log_10_Gata3^mit-stim^ | *F*_1, 367.9_ = 16.07 | <0.001 | Lesion^+^ 5.025 ± 1.254 |
