# Supplementary material for: An Immunological Marker of Tolerance to Infection in Wild Rodents
Source: PLoS Biol. 2014 Jul 8;12(7):e1001901. doi: 10.1371/journal.pbio.1001901 (PMC4086718; doi:10.1371/journal.pbio.1001901)
Supplement: Table S11 — Associations between parasitic infections and mitogen-stimulated Gata3 expression (Gata3mit-stim) in adult males (cross-sectional study). The association of Gata3mit-stim with grouped parasitic infection was analyzed in LMMs of the form: Parasite variable = Process group+SVL+Log10 Gata3mit-stim (random term = Year×Sampling Point×Site). In mature males there were significant positive associations with both of the macroparasite principal component variables (PCM main and PCM) (see table). When immature males were added to the above model for PCM main, with different age-specific slopes, there was significant variation in the Gata3mit-stim slopes, with a positive slope for mature males and a negative slope for immature males (see main text, Figure 1C) (Stage×Gata3mit-index interaction, F 1,204.2 = 16.3, p = 7.6×10^−5^). A similar interaction also occurred in the case of PCM (p = .031). Significant positive association in main hypothesis test is highlighted in orange; significant association in post hoc test is highlighted in yellow. (DOC) [file pbio.1001901.s016.doc]

| **Variable** | **Test statistic** | ***P*** | **Parameter ± standard error** |
| --- | --- | --- | --- |
| **PC^M main^** | ***F*_1, 130.6_** = **7.47** | **0.007** | **0.5565 ± 0.2036** |
| **PC^M^** | ***F*_1, 124.05_** = **4.58** | **0.034** | **0.4461 ± 0.2085** |
