# Supplementary material for: An Immunological Marker of Tolerance to Infection in Wild Rodents
Source: PLoS Biol. 2014 Jul 8;12(7):e1001901. doi: 10.1371/journal.pbio.1001901 (PMC4086718; doi:10.1371/journal.pbio.1001901)
Supplement: Table S12 — Instantaneous and time-lagged associations between exposure to blood-feeding ectoparasites and Gata3 expression (Gata3blood) in peripheral blood in adult males (longitudinal study). (A) Time lagged associations. Association of Gata3blood with infection variables earlier in time (t-1 = 1 mo earlier). When analyzed alone, ticks and small fleas were represented by continuous log-transformed abundance variables. When ticks and small fleas were analyzed together, this was as a binary presence/absence factor (0, no infection; 1, infection with ticks and or fleas). Table shows significant explanatory terms from models of the form: Gata3blood = Weight+Parasite variable (random terms: Year×Sampling point×Site+Assay plate+Individual ID). Results for weight are for the model with a factor for ticks and or small fleas. Significant positive associations highlighted in yellow and significant negative associations in grey. Earlier infection tended to be positively associated with Gata3blood expression. (B) Instantaneous association. Association of Gata3blood with infection at the same time point. When analyzed alone, ticks and small fleas were represented by continuous log-transformed abundance variables. When ticks and small fleas were analyzed together this was as a binary presence/absence factor (0, no infection; 1, infection with ticks and or fleas). Table shows significant explanatory terms from models of the form: Log10 Gata3blood = Weight+Parasite variable (random terms: Year×Sampling Point×Site+Assay plate+Individual ID). Results for weight are for the model with a factor for ticks and or small fleas. Significant (or marginally nonsignificant) negative associations highlighted in grey. Contemporaneous infection tended to be negatively associated with Gata3blood expression, perhaps due to some protective (resistance) effect of this response. (DOC) [file pbio.1001901.s017.doc]

**A. Time lagged associations.**

**B. Instantaneous association.**

| **Term** | **Test statistic** | P | **Parameter ± standard error** |
| --- | --- | --- | --- |
| **Weight** | ***F*_1, 151.5_ = 8.37** | **0.004** | **-0.0145 ± 0.0050** |
| **Ticks (t-1)** | ***F*_1, 95.8_ = 5.03** | **0.027** | **0.0094 ± 0.0042** |
| Small fleas (t-1) | *F*_1, 102.0_ = 2.12 | 0.149 | 0.0380 ± 0.0261 |
| **Ticks and or small fleas (t-1)** | ***F*_1, 103.2_ = 6.89** | **0.010** | **0.1679 ± 0.0640** |
| **Earlier exposure to ticks and or small fleas (all prior records)** | ***F*_1, 118.5_ = 8.55** | **0.004** | **0.2258 ± 0.0773** |

| **Term** | **Test statistic** | P | **Parameter ± standard error** |
| --- | --- | --- | --- |
| **Weight** | ***F*_1, 227.5_ = 19.98** | **1.2 × 10^-5^** | **-0.0056 ± 0.0015** |
| **Ticks** | ***F*_1, 332.0_ = 6.02** | **0.015** | **-0.0029 ± 0.0012** |
| Small fleas | *F*_1, 275.4_ = 1.05 | 0.307 |  |
| **Ticks and or small fleas** | ***F*_1, 331.2_ = 3.46** | **0.064** | **-0.0537 ± 0.0288** |
