# Supplementary material for: An Immunological Marker of Tolerance to Infection in Wild Rodents
Source: PLoS Biol. 2014 Jul 8;12(7):e1001901. doi: 10.1371/journal.pbio.1001901 (PMC4086718; doi:10.1371/journal.pbio.1001901)
Supplement: Table S13 — Time-lagged association between month-on-month weight gain and Gata3 expression (Gata3blood) in peripheral blood in adult males (longitudinal study). Association of month-on-month weight gain (adjusted for starting weight) with Gata3blood expression 1 mo earlier. Table shows significant explanatory terms from LMMs of the form: Final weight = Starting weight+Gata3blood (random terms: Year×Sampling Point×Site+Individual ID). Gata3blood was positively associated with adjusted weight gain in the following month. There was no association between Gata3blood and weight gain in the preceding month (analyzed in an equivalent model). The effects of individual parasite variables were examined in these base models (taking each variable in turn), but were nonsignificant. Macroparasite variables examined included the abundance of fleas, ticks, laelapid mites, listrophorid mites, and lice and also an overall ectoparasite index; microparasite variables (presence/absence) included B. microti, Bartonella spp., and overt TB. Ectoparasite index was an additive score based on the sum of standardized abundances for the different ectoparasites. Significant positive associations are highlighted in yellow. (DOC) [file pbio.1001901.s018.doc]

| **Term** | **Test statistic** | P | **Parameter ± standard error** |
| --- | --- | --- | --- |
| **Starting weight** | ***F*_1, 167.4_ = 440.79** | **<5 × 10^-7^** | **0.7961 ± 0.0379** |
| **Gata3^blood^** | ***F*_1, 155.7_ = 4.33** | **0.039** | **1.250 ± 0.600** |
