# Supplementary material for: An Immunological Marker of Tolerance to Infection in Wild Rodents
Source: PLoS Biol. 2014 Jul 8;12(7):e1001901. doi: 10.1371/journal.pbio.1001901 (PMC4086718; doi:10.1371/journal.pbio.1001901)
Supplement: Table S14 — SEM of key variables from cross-sectional study. In the SEM analysis of the cross-sectional data, we searched for models with: the causality indicated by the longitudinal analyses in Tables S12 and S13; higher numbers of exclusively significant (p<.05) or near-significant (.05<p<.08) parameters; and optimal model fit (root mean square error of approximation, RMSEA) and AIC statistics. The table shows the two best supported models with six or seven significant (or near-significant) parameters. Parameter estimates for model 2 are shown in Table S15. †Pattern of dependency assumed in each model. Gr, overall growth rate (SVL adjusted for age estimated by lens weight); P, infection with key influential macroparasites (PCM main, see Table S1); G, Log10 Gata3 expression in mitogen-stimulated splenocytes; C, body condition; T, testis condition. Prior to the SEM analysis, all of the variables were adjusted, in general linear models, for spatiotemporal sampling point (sampling time and site nested within year) and for host linear dimensions (SVL+SVL2) if SVL did not contribute to the variable already. (DOC) [file pbio.1001901.s019.doc]

| **Model** | **Terms†** | **AIC** | ***P* RMSEA <0.05** | ***Χ*^2^** | **DF** | ***P*** |
| --- | --- | --- | --- | --- | --- | --- |
| **1** | Gr ← P  G ← P + T  C ←P + T + G | 1804 | 0.52 | 1.89 | 2 | 0.389 |
| **2** | Gr←P  G ← P  C←G + P  T ←P + C + G | 1807 | 0.81 | 0.604 | 2 | 0.739 |
