# Supplementary material for: An Immunological Marker of Tolerance to Infection in Wild Rodents
Source: PLoS Biol. 2014 Jul 8;12(7):e1001901. doi: 10.1371/journal.pbio.1001901 (PMC4086718; doi:10.1371/journal.pbio.1001901)
Supplement: Table S15 — Parameter estimates for SEM best supported by independent temporal analyses of longitudinal data. Gr, overall growth rate (SVL adjusted for age); P, infection with key influential macroparasites (PCM main, see Table S1); G, Log10 Gata3 expression in mitogen-stimulated splenocytes; C, body condition; T, testis condition. Prior to the SEM analysis, all of the variables were adjusted, in general linear models, for spatiotemporal sampling point (sampling time and site nested within year) and for host linear dimensions (SVL+SVL2) if SVL did not contribute to the variable already. A causal effect of macroparasites (P) upon Gata3 expression (G) and of G upon body condition (C) was independently suggested by analyses of the longitudinal dataset (see respectively, Tables S12 and S13). The stimulation of mammalian Th2 responses (including Gata3 expression) is also a well-known effect of macroparasite infection in experimental settings (see main article). (DOC) [file pbio.1001901.s020.doc]

| **Dependent variable** | **Influencing variables** | **Parameter estimate (± s.e.)** | ***Z*-value** | **P** |
| --- | --- | --- | --- | --- |
| Gr ← | P | -0.87 ± 0.43 | -2.05 | 0.041 |
| G ← | P | 0.10 ± 0.031 | 3.32 | 0.001 |
| C ← | G | 2.52 ± 0.68 | 3.71 | <0.0005 |
|  | P | 0.64 ± 0.27 | 2.36 | 0.018 |
| T ← | G | -0.064 ± 0.018 | -3.57 | <0.0005 |
|  | P | 0.014 ± 0.007 | 1.91 | 0.056 |
|  | C | 0.005 ± 0.002 | 2.64 | 0.008 |
