# Supplementary material for: An Immunological Marker of Tolerance to Infection in Wild Rodents
Source: PLoS Biol. 2014 Jul 8;12(7):e1001901. doi: 10.1371/journal.pbio.1001901 (PMC4086718; doi:10.1371/journal.pbio.1001901)
Supplement: Table S16 — Gata3 expression in peripheral blood (Gata3blood) and age-specific primary session return rate (longitudinal study). Return rate of mature males (at the next primary trapping session) (A) was initially analyzed with respect to Gata3 expression in peripheral blood (Gata3blood) using a GLMM with binomial errors (trial size = 1). The base model was of the form: Return∼Weight+Gata3blood+Peripheral row (random term = Year×Sampling Point×Site+Individual ID). The peripheral row factor accounted for animals captured in the outer trap row of the grid, which might putatively have lower recapture probabilities (as their ranges are more likely to have been centred outside the grid). Other models (not shown) examined the effects of individual parasite variables in this base model (taking each in turn). Macroparasite variables included the abundance of fleas, ticks, laelapid mites, listrophorid mites, and lice and also an overall ectoparasite index; microparasite variables (presence/absence) included B. microti, Bartonella spp., and overt TB. Ectoparasite index was an additive score based on the sum of standardized abundances for the different ectoparasites. These were only included in the final model if significant. A similar set of models was then investigated (post hoc) for all stages (B), additionally including life history stage as a factor. The main effect for Gata3blood was not significant in adult males but was significantly negative in the analysis across all stages (p = .003, F 1, 863.7 = 9.02, parameter −0.5852±0.1949). Significant Weight×Gata3blood interactions occurred in both analyses (with Gata3blood having an increasingly positive effect on survival as hosts became heavier). Although return rate might act as a reasonable surrogate for survival (given the high recapture rates observed (see Table S17)), further analyses explicitly modelled survival in the context of variable recapture probability (Table S17). Significant positive associations in tables A–B are highl [file pbio.1001901.s021.doc]

A. Adult males

| **Term** | **Test statistic** | P | **Parameter ± standard error** |
| --- | --- | --- | --- |
| Peripheral row | *F*_1, 321.7_ = 0.01 | 0.914 |  |
| **Weight.Gata3^blood^** | ***F*_1, 336.4_ = 4.41** | **0.036** | **0.0738± 0.0350** |

**B. All stages**

| **Term** | **Test statistic** | P | **Parameter ± standard error** |
| --- | --- | --- | --- |
| **Ectoparasite index** | ***F*_1, 892.4_ = 4.05** | **0.044** | **-0.0766 ± 0.0358** |
| **Peripheral row** | ***F*_1, 912.2_ = 3.46** | **0.063** | **-0.3044 ± 0.1636** |
| **Stage** | ***F*_10,887.3_ = 28.96** | **0.001** |  |

| **Weight.Gata3^blood^** | ***F*_1, 878.2_ = 5.51** | **0.011** | **0.0487 ± 0.0191** |
| --- | --- | --- | --- |
