# Supplementary material for: An Immunological Marker of Tolerance to Infection in Wild Rodents
Source: PLoS Biol. 2014 Jul 8;12(7):e1001901. doi: 10.1371/journal.pbio.1001901 (PMC4086718; doi:10.1371/journal.pbio.1001901)
Supplement: Table S17 — Gata3 expression and survival: CJS analysis with time-specific individual covariates (longitudinal study). The effect of Gata3 expression in peripheral blood (Gata3blood) was analyzed in CJS models in a subset of males with >1 capture and no or few missing values for Gata3blood (n = 107). (This tended to bias the analysis towards larger males due to growth occurring in the capture intervals.) Time-specific body weight covariates were highly significant (LRT, χ2 = 17.25, DF = 5, p = .004) when added to an optimal base model for survival and recapture that included seasonal (monthly) variation in survival (φ) and variation across years in recapture probability (p). Gata3blood and Gata3blood×Body Weight covariates were then added to the base model already containing body weight covariates. The best resulting models contained covariates for Gata3blood and Gata3blood×Body Weight only in the last 2 mo (LRT, χ2 = 16.49, DF = 4, p = .0024) or last month of the season (LRT, χ2 = 15.37, DF = 2, p = .0005). In these months, survival tended to increase in larger animals expressing a greater amount of Gata3blood. The following parameter values were estimated for a model in which overall p and φ for the first time interval (φ April→May) were constrained to be 1. This was because p was generally estimated to be very high (>0.8–0.9 in models with constant, year-on-year, or seasonal variation in p) and because φ April→May was limited by the nature of the data, which only included animals with multiple recaptures. Real function parameters for survival were: φ May→June = 0.49±0.07, φ June→July = 0.76±0.06, φ July→August = 0.87±0.05, φ August→September = 0.73±0.05, φ September→October = 0.55±0.08. The logit link function parameter for the September Gata3blood×Body Weight covariate was: βSeptember = 0.3±0.11. (DOC) [file pbio.1001901.s022.doc]

| **Model** | **AICc** | **ΔAICc** |
| --- | --- | --- |
| *φ* **month + weight + Gata3^blood^** _September_ **+ Gata3^blood^ × weight** _September_  ***p* year** | **390** |  |
| *φ* **month + weight *p* year** | **401** | **11** |

.
