# Supplementary material for: An Immunological Marker of Tolerance to Infection in Wild Rodents
Source: PLoS Biol. 2014 Jul 8;12(7):e1001901. doi: 10.1371/journal.pbio.1001901 (PMC4086718; doi:10.1371/journal.pbio.1001901)
Supplement: Table S18 — Real-time PCR primers used to measure the expression of immunological genes in peripheral blood. (DOC) [file pbio.1001901.s023.doc]

| Gene | Primer sequence | Role in assay | |
| --- | --- | --- | --- |
|  |  |  | |
| Sdha | L 5’-GGCTGATCGAACAGGCCATT-3’  R 5’-GCATTCCCCACTTTCCATCAA-3’ | | Endogenous control |
| Ywhaz | L 5’-AAAAAGGAGATGCAGCCGACA-3’  R 5’-AGCAGGCTTTCTCTGGGGAGTT-3’ | | Endogenous control |
| Gata3 | L 5’-ACCTCCTGTGCGAACTGTCAGA-3’  R 5’-TGCAGCTTGTAGTAGAGCCCACA-3’ | | Target |
| IL-10 | L 5’-AACCACGGCCCAGAAATCAA-3’  R 5’-CACAGGGGAGAAATCGATGACA-3’ | | Target |
| IFN-γ | L 5’- GGTCCAACGCAAAGCTGTCA-3’  R 5’- GCCGATTTCCAACAGCGAAA-3’ | | Target |
