# Supplementary material for: An Immunological Marker of Tolerance to Infection in Wild Rodents
Source: PLoS Biol. 2014 Jul 8;12(7):e1001901. doi: 10.1371/journal.pbio.1001901 (PMC4086718; doi:10.1371/journal.pbio.1001901)
Supplement: Table S19 — Infection statistics for parasites at Kielder broken down by Locality×Year of Study. A, abundance±standard error. P, prevalence, followed by 95% CI. *Abundance data for Listrophoridae relate to a semiquantitative abundance index. †PCR diagnosis of Bartonella spp. and B. microti may have had unequal sensitivities in 2008 and 2009 due to the use of different DNA extraction methods. Prevalence data are thus not directly comparable across years. (DOC) [file pbio.1001901.s024.doc]

|  | **BLB 2008-9** | | **SQC 2008-9** | | **SCP 2009-10** | | **KTH 2009-10** | |
| --- | --- | --- | --- | --- | --- | --- | --- | --- |
|  | **A** | **P** | **A** | **P** | **A** | **P** | **A** | **P** |
| Larval cestodes (*Taenia* spp.) | 0.01±0.01 | 1, 0-4 | 0.04±0.02 | 3, 1-7 | 0 | 0 | 0.04±0.02 | 4, 2-9 |
| Adult cestodes | 1.68±0.27 | 48, 40-56 | 1.02±0.12 | 48, 40-57 | 1.03±0.16 | 44, 35-53 | 0.96±0.17 | 38, 29-46 |
| *Heligmosomoides laevis* | 0.08±0.06 | 2, 0-6 | 0.05±0.02 | 3, 1-6 | 0.24±0.10 | 10, 5-16 | 0.07±0.03 | 4, 2-9 |
| *Trichurus arvicolae* | 0.07±0.04 | 4, 1-8 | 0.01±0.01 | 1, 0-4 | 0.02±0.02 | 1, 0-4 | 0.01±0.01 | 1, 0-5 |
| *Syphacia nigeriana* | 3.32±0.93 | 22, 16-30 | 1.99±0.76 | 14, 9-20 | 3.9±1.17 | 30, 23-39 | 2.96±1.02 | 29, 21-37 |
| Ticks (*Ixodes* spp.) | 1.98±0.82 | 28, 21-35 | 1.05±0.27 | 19, 13-26 | 1.51±0.50 | 32, 24-40 | 2.08±0.56 | 39, 31-48 |
| Myobiidae | 0.55±0.30 | 4, 1-8 | 3.48±2.41 | 8, 4-13 | 1.84±0.69 | 15, 10-22 | 3.76±2.31 | 16, 10-23 |
| Laelapidae | 2.38±0.28 | 53, 44-61 | 2.91±0.34 | 63, 54-70 | 3.30±0.44 | 68, 60-76 | 3.57±0.59 | 53, 44-62 |
| Listrophoridae* | 1.55±0.09 | 76, 68-82 | 1.68±0.08 | 83, 76-88 | 1.36±0.10 | 67, 59-75 | 1.36±0.09 | 73, 65-80 |
| Ear mites | 0 | 0 | 0 | 0 | 0.04±0.04 | 1, 0-4 | 0.13±0.08 | 3, 1-7 |
| *Hoplopluera acanthopus* | 0.14±0.08 | 5, 2-9 | 0.04±0.02 | 2, 0-6 | 0.21±0.12 | 4, 1-9 | 0.54±0.2 | 7, 4-13 |
| Small fleas | 1.60±0.19 | 53, 45-61 | 0.97±0.11 | 48, 40-57 | 1.45±0.19 | 53, 43-60 | 2.99±0.38 | 65, 56-73 |
| *Hystrichopsylla talpae talpae* | 0.17±0.04 | 14, 9-21 | 0.15±0.04 | 12, 8-18 | 0.09±0.03 | 9, 5-15 | 0.08±0.03 | 6, 3-11 |
| *Bartonella* spp.† | - | 11, 6-17 | - | 22, 16-29 | - | 57, 46-67 | - | 57, 47-67 |
| *Babesia microti*† | - | 25, 18-33 | - | 19, 14-27 | - | 35, 25-46 | - | 22, 14-32 |
| Overt TB | - | 4, 1-8 | - | 1, 0-5 | - | 1, 0-5 | - | 3, 1-7 |
