# Supplementary material for: An Immunological Marker of Tolerance to Infection in Wild Rodents
Source: PLoS Biol. 2014 Jul 8;12(7):e1001901. doi: 10.1371/journal.pbio.1001901 (PMC4086718; doi:10.1371/journal.pbio.1001901)
Supplement: Methods S1 — Supplementary Methods. (DOC) [file pbio.1001901.s025.doc]

**Methods S1**

**1.1. Study sites**

Study sites monitored in 2008-2009 were designated SQC (55.2549, -2.6116; cross-sectional *n* = 155) and BLB (55.2457, -2.6108; *n* = 152) and sites monitored in 2009-2010 were designated SCP (55.2638, -2.5458, *n* = 132) and KTH (55.2616, -2.6171, *n* =136). Each of these sites was a clear-cut area prominently colonized by *Deschampsia caespitosa, Agrostis capillaris* and *Juncus effusus*, occurring adjacent to commercial coniferous forests, uninhabitable by field voles, dominated by Sitka Spruce (*Picea sitchensis*). All sites represented discrete patches of habitat enclosed by forest trees, metalled forestry tracks, tarmac roads and/or semi-permanent water courses.

**1.2. Live-trapping**

All live-trapping was carried out using Ugglan Special Mousetraps (Grahnab, Marieholm, Sweden) pre-baited 3 days before each trapping session. Grid traps were placed in permanent (pegged) stations in runs and feeding sites used by voles (as indicated by field signs). Each monthly trapping session (primary session) consisted of 5 consecutive checks (morning/evening/morning/evening/morning) of continuously set traps. Transect traps at each site were set on the last day of the grid trapping session only. In between trapping sessions all traps were opened and left *in situ*. A total of 960 animals were marked and 1665 captures or recaptures recorded during the longitudinal study.

**1.3. Cross-sectional study**

**1.3.1. Animal handling and host biometric data**

Details of animal handling and the collection of biometric data are described in Jackson *et al.* 2011 (S*1*). Monthly transect samples from each site were returned to the laboratory, weighed and housed in identical individual cages under a lighting regimen equivalent to the natural photoperiod at the time of capture. Each cage had soft wood chippings on its floor and contained sufficient soft bedding for the animal to make a nest. Each animal had free access to fresh water, bruised oats and carrot. Approximately half of each group of animals was processed between 08.00 and 11.00 on the next day (25-28 hours post-capture). The remainder of the group was processed at the same time on the following day (49-52 hours post capture). Each animal was killed by an overdose of chloroform followed by exsanguination. Snout to vent length (SVL, mm) and body weight (g) were recorded prior to exsanguination. Each animal was then dissected in a Class 2 safety cabinet and the spleen was removed to RPMI 1640. Liver and spleen wet weights (g) and reproductive status were recorded. Individual hosts were classified in the following life history stage (LH) categories. Males, 1: non-scrotal with undeveloped testes (i.e., in non-mating condition), 2: scrotal with large testes and expanded seminal vesicles (i.e., mating condition); females, 1: imperforate, non-lactating, non-pregnant (i.e., non-mating), 2: perforate, non-pregnant, non-lactating (i.e., mating), 3: pregnant.

**1.3.2. Cell culture**

Cell culture methods used for animals in the cross-sectional study are described in Jackson *et al.* 2011 (S*1*). Spleens were disaggregated through a 70μm cell strainer into RPMI 1640. Following eythrocytic lysis (Sigma R7757), leucocytes were washed three times in RPMI 1640 and then cultured (37^o^C, 5% CO_2_) on 96 well plates at 2 × 10^6^ cells/ml in RPMI 1640 supplemented with 24mM Na HCO_3_, 10% heat inactivated foetal calf serum, 2mM L-glutamine, 100u/ml penicillin, 100µg/ml streptomycin and 60μM monothioglycerol. For 24 h assays, triplicate individual cultures (300 μl volume) were stimulated with the TLR2 agonist HKLM (heated-killed *Listeria monocytogenes* cells at 1 x 10^8^ cells ml^-1^) or the synthetic TLR7 agonist imiquimod (at 20 μg ml^-1^) or left un-stimulated. Cells were harvested after 24 h and stored in RNAlater (Ambion) at -80^o^C. All TLR agonist reagents utilized were tested by the manufacturer (InvivoGen) for functional induction of the target TLR and for endotoxin contamination. For 96 h assays, quadruplicate individual 300μl cultures were stimulated with the mitogen phytohaemagglutinin (PHA-L) at 40 μg ml^-1^ or left un-stimulated. Cells were harvested after 96 h and stored in RNAlater at -80^o^C. For each assay type a single culture plate was used for all animals on each process day. Stimulant concentrations used in assays were chosen to produce maximal expression levels of pro-inflammatory mediators at the given time points, based on preliminary dose-response experiments with splenocyte cultures from Kielder *M. agrestis*. For the 96 h assay conditions, time course experiments in a subset of Kielder voles indicated that, following PHA stimulation, there was an initial peak for IFN-γ expression at 12-48 h, with a later peak at 72-120 h. As the early peak may have related to innate responses, a later time point (96 h) was chosen for these assays in order to correspond to an expected expansion of CD4^+^ T-helper cells (S*2*) in stimulated cells.

**1.3.3. Splenocyte immune expression measurements**

**1.3.3.1. *24 h TLR assay.*** As described in Jackson *et al.* (2011) (S*1*), we measured mRNA accumulations for a set of immune system genes in 24 h splenocyte cultures stimulated with agonists for the cell surface Toll-like receptor (TLR) TLR2 or the endosomal receptor TLR7, and also in un-stimulated control cultures. The genes measured were the pro-inflammatory cytokine IL -1β, IRF5 (a transcription factor regulating TLR-mediated production of type I interferons and pro-inflammatory cytokines (S*3, 4*)) and the regulatory cytokines TGF-β1 (S*5*) and IL-10 (S*6*).

**1.3.3.2. *96 h mitogen assay.*** As described in Jackson *et al.* (2011) (S*1*), we measured mRNA accumulations for a set of immune system genes in 96 h splenocyte cultures stimulated with PHA-L and also in un-stimulated control cultures. The genes measured were the Th1 inflammatory cytokine IFN-γ, Tbet (a transcription factor associated with the Th1 cell subset (S*7*)), Gata3 (a transcription factor associated with the Th2 cell subset (S*8, 9*)), FoxP3 (a transcription factor associated with regulatory T-helper cells (S*10, 11*)) and the regulatory cytokines TGF-β1 and IL-10 (S*12*).

**1.3.4. Parasitological measurements**

**1.3.4.1. *Macroparasites.*** We recorded 25 species of macroparasites at the study localities in Kielder. For practical reasons and analytical tractability some species were grouped into phylogenetically and ecologically coherent categories. All intestinal cestodes (*Anoplocephaloides dentata* aff., *Paranoplocephala* sp., *Rodentolepis asymmetrica*, *Arostrilepis horrid*a) occurring at the adult stage in field voles were included in a group (adult cestodes), whilst taeniid cestodes (*Taenia taeniaeformis*, *Taenia mustelae*, *Taenia polyacantha*) occurring at the larval stage in field voles were included in another group (larval cestodes). Myobiid fur mites, listrophorid fur mites, laelapid mites, ticks (*Ixodes* spp.), lice (*Hoplopleura acanthopus*) and smaller flea species (*Ctenophthalmus nobilis*, *Peromyscopsylla spectabilis*, *Megabothris walkeri*, *Malaraeus penicilliger*, *Rhadinopsylla pentacantha*), were also respectively included within single categories. Other species recorded individually were the large, slow-moving flea *Hystrichopsylla talpae talpae* and three gastrointestinal nematodes: the trichinelloid, *Trichuris arvicolae*; the trichostrongyloid, *Heligmosomoides laevis*; and the oxyuroid, *Syphacia nigeriana*. Direct counts of individuals were recorded for all species (or groups of species) except in the case of listrophorid mites, for which a semi-quantitative abundance index (0-3) was used due to the very high number of individuals. Basic summary infection data for these taxa across sites are shown in Table S19.

**1.3.4.2. *Microparasites.*** We recorded overt symptoms of vole tuberculosis (TB) caused by *Mycobacterium microti* infection (cutaneous lesions and subcutaneous nodules) (S*13*). We also determined PCR positivity of *Babesia microti* and of *Bartonella* spp. in cardiac blood samples (collected during exsanguination, see above). In the 2008-2009 season, blood samples were dried onto filter paper and then DNA extracted using a protocol adapted from Bereczky *et al.* (2005) (S*14*). In the 2009-2010 season samples were stored frozen and DNA extracted by the alkaline digestion method described by Bown *et al.* (2003) (S*15*). *Bartonella* spp. were detected with universal (*Bartonella* genus-specific) primers by the PCR protocol described in Telfer *et al.* (2005) (S*16*) and *B. microti* by the real-time PCR protocol described in Bown *et al.* (2008) (S*17*).

**1.4. Longitudinal study**

**1.4.1. Animal handling, biometric data and samples**

Following first capture within the study, each animal was marked with an AVID transponder. On first capture within a primary trapping session individual animals were weighed with a spring balance (Pesola) and the sex and life history stage determined. Males were determined as scrotal or non-scrotal (with or without descended testes). The reproductive stage of females was inferred from the perforation of the vulva, the degree of pelvic closure, the development of the nipples and the degree of abdominal distension (which can indicate advanced pregnancies). A peripheral blood sample (< 50µl) was taken from the tail tip and placed directly in 500 µl of RNAlater. Microtubes containing the blood-RNAlater samples were stored temporarily at -20^o^C and then transferred to -80^o^C within one week.

## 1.4.2. Parasitological measurements

**1.4.2.1. *Macroparasites.*** The full body surface of each animal was examined for the presence of small fleas, mole fleas, myobiid mites, laelapid mites, listrophorid mites and ixodid ticks. Direct counts of individuals were made for ticks and mole fleas. Semi-quantitative abundance indices were recorded for myobiid mites, laelapid mites, listrophorid mites and small fleas.

**1.4.2.2. *Microparasites.*** An aliquot of the pelleted RNA-later preserved blood sample (~ 10 µl) from each animal was removed for the PCR diagnosis of *Babesia microti* and *Bartonella* spp. This blood was mixed (1:4) in lysis buffer (4M guanidium thiocyanate acid, 0.5% N-lauroyl sarcosine, 1 mM dithiothreitol (DTT), 25 mM sodium citrate) and glycogen added to a final concentration of 100 µg ml^-1^. Following precipitation in isopropyl alcohol and one wash in ethanol, the dried DNA pellet was re-suspended in nuclease free water. Using these DNA samples as PCR templates, *Bartonella* spp. and *B. microti* were detected as above. Overt symptoms of TB were recorded in the field (S*13*).

1.4.3. Peripheral blood immune expression measurements

Methods for the measurement of gene expression in peripheral blood samples from continuously monitored animals are those used by Jackson *et al.* (2011) (S*1*) for splenocytes, except in the following details. RNA was extracted from blood samples stored in RNAlater using Mouse Ribopure^TM^ blood RNA isolation kits (AM1951, Ambion). New real-time PCR primers (Table S18) were designed (with amplicons ~100bp) using Primer Express® (version 3.0) software, as efficiencies of the original primer sets (used for splenocyte-derived cDNA) were suboptimal in the blood-derived cDNA samples. A new housekeeping gene stability analysis of the 7 candidate genes trialled by Jackson *et al.*, 2011 (S*1*) was carried out on 10 randomly selected field blood samples, identifying Ywhaz and Sdha as the most suitable endogenous controls. Only three targets, IFN-γ, Gata3 and IL-10 were measured (respectively representing Th1, Th2 and regulatory responses), normalizing to both Ywhaz and Sdha (using the arithmetic mean of their C_T_ values).

# 1.5. Data analysis

**1.5.1. Overall strategy**

Our analytical strategy is summarized in Figure S4 and the statistical techniques used are described in detail in sections 1.5.2-3. In analysing our dataset we initially aimed to find a pattern of host resistance or tolerance to infection, considering both the dataset as a whole and when stratified by life history stage. For this we used the data from the cross-sectional hosts (from which more precise and exhaustive infection measurements were available) (see section 1.5.2).

As discussed in the main article, resistance is the tendency for a host to prevent invasion by, or expel, parasites; a pattern of tolerance is the tendency of a host to accumulate parasites whilst limiting the damage caused thereby. As one pattern that might be indicative of acquired resistance we searched for non-linear associations of parasite infection with biometric proxies for host age (dry eye lens weight or snout-vent length). Thus, acquired immune responses (usually the main player mediating resistance in co-adapted host parasite systems) would be expected to increase with age in hosts expressing resistance, resulting in a decelerating positive, convex or negative association with parasite burden. As another indicator of resistance, we also considered the possibility of negative associations between macroparasite infection and the expression of Gata3 (a transcription factor involved in Th2 immunity, which is usually associated with protection against macroparasites).

As a pattern that might be indicative of tolerance we searched for association between host condition indices and parasite infection: relating the residual of body and organ weights on SVL and its quadratic term (a size adjusted condition index) to parasite abundance in individual hosts. Thus, individuals that maintained a better condition relative to parasite load might be expected to be expressing more of the processes associated with tolerance. Furthermore, the stratification of this analysis by life history group allowed the slopes of host condition on parasite load to be interpreted as differing group-specific reaction norms. These slopes might be referred to as “epidemiological reaction norms” (ERNs) to distinguish them from reaction norms generated under experimental conditions and to acknowledge the possibility of confounding influences in the present form of study (see below for our approach to possible confounding influences).

Where life history groups showed particularly divergent patterns of resistance (differing slopes of infection on age indicators and resistance markers) and tolerance (differing slopes of condition on parasite load), we focussed upon these stages in subsequent analyses.

Following the identification of patterns suggestive of a tolerance or resistance strategy in particular host strata, we then intended to relate these to individual-level variations in immunological gene expression measurements: to find immunological markers. Then, in turn, we aimed to relate any immunological markers to their life-history consequences: analyzing their association with biometric data reflecting life history traits and their effect on survival patterns (in the longitudinal data).

In order to avoid errors of interpretation due to multiplicity we adopted the following approach. We initially employed principal components analysis (PCA) to reduce our cross-sectional parasitological and immunological variable sets (where there was useful redundancy) to a smaller number of non-redundant summary variables that were then used in sparing initial main hypothesis tests. Subsequent testing was carried out in a sequential manner. Each significant main hypothesis test was followed by *post-hoc* testing of individual variables (to indentify individual variables of major interest) and by the formulation of a further main hypothesis to be addressed in the next round of testing (see Figure S4). Where a single non-redundant variable of major interest was identified by the *post-hoc* testing at a given point in the analysis (e.g., mitogen-stimulated Gata3 responses amongst the immunological variables) this became the focus of later hypotheses and replaced the corresponding reduced variable in main hypothesis tests in later analyses. Multiplicity adjustments (sequential Bonferroni) were applied for the main hypothesis tests at each stage of the analysis, if there was more than one test.

In order to account for possible confounding in our analyses of the cross sectional data (where the direction of causality is often uncertain, or there may be unmeasured causal variables) we used focussed temporal analyses of our longitudinal (live-trapping) dataset to independently infer the existence and direction of causality (where this was hypothesized following initial analysis of the cross-sectional data). Thus, under the presumption that cause precedes effect, we were able to suggest causal and dependent variation.

Finally, using structural equations modelling (SEM), we examined simultaneous patterns of interdependency in the main cross-sectional variables of interest, using our longitudinal analyses (see above) to help select candidate causal hypotheses.

# 1.5.2. Cross-sectional data

Macroparasite data consisting of individual counts and all immunological variables were log-transformed (log_10_ [*x* +1]) prior to the following analyses.

We used PCA (see Table S1) to provide grouped measures of macroparasite abundance that were used in all main hypothesis tests. The relatively small number of microparasite variables (*B. microti*, *Bartonella* spp. and overt TB) were analyzed separately. We also applied PCA to provide grouped measures of immune responses (see Table S2), these being used in all main hypothesis tests up to the point where individual variables of major effect were identified.

To search for patterns of acquired resistance, parasite variables were analyzed as the response with respect to SVL or lens weight (and their quadratic terms) in linear mixed models (LMMs) for continuous macroparasite variables and in generalized linear mixed models (GLMMs) with binomial errors for presence/absence microparasite variables.

To search for patterns indicative of tolerance, host body and organ weights were analyzed as the response in LMMs with respect to parasite variables. These LMMs additionally included SVL and its quadratic term in the fixed element to allow interpretation in terms of a condition index (i.e., high or low weight relative to linear dimension). Details of these models are given in Tables S3-S6.

To search for associations between host condition (body and organ weights adjusted for SVL and its quadratic term) and immune expression variables, the immune expression variables were added as covariates to LMMs of the above form, retaining a significant term for grouped macroparasite infection. Details of these models are given in Tables S8-S9. LMMs were also used to investigate some hypothesized associations between immune expression and macroparasite infection (details of model in Table S11).

Structural equations modeling (SEM) (S*18*) was then employed to examine patterns of dependency amongst a suite of variables implicated in significant associations (in adult males) in the above analyses (for details see Table S14). Prior to the SEM analysis, data were adjusted for variation due to body size and spatiotemporal sampling point in general linear models (GLMs) with explanatory terms for SVL + SVL^2^ (where SVL did not contribute to a variable already) and for sampling time and site, respectively nested within year. Results from our analyses of longitudinal data (section 1.5.3) were used to select candidate causal hypotheses in this analysis.

# 1.5.3. Longitudinal data

Analyses of temporal sequences in presence/absence or continuous variables from the longitudinal data set were respectively carried out with GLMMs (binary errors) and LMMs. The fixed element of these models was used to represent biometric and infection variables and the random element to represent individual identity, the time and place of sampling and, in cases where immune expression variables were the response, the Q-PCR assaying structure (i.e., Q-PCR plate). Details for specific analyses are provided in Tables S12-S13.

Initial analyses of survival patterns (in all stages and in mature males) used GLMMs (binary errors) with capture at next primary trapping session as the response. This assumed that recapture probability of surviving animals was high within primary sessions, which was supported by Cormack-Jolly-Seber (CJS) analyses of survival and recapture probability (see Table S17).

A further analysis of male survival was carried out in a CJS framework (S*19, 20*), explicitly estimating both survival (*φ*) and recapture rates (*p*) from the return rates of individually marked animals. Only males with total primary session returns ≥1 (to avoid transient individuals) and covariate data (weight and Gata3^blood^) were included in the analysis, biasing it towards larger animals. Missing covariate values within a sequence of captures for an individual animal were assigned the mean of the flanking values. For occasions when an animal was not captured, covariate values were set at the time-specific variable mean. The February and March trapping sessions in 2008 (trapping started in April 2009) were discarded so that the data could be analyzed in the same model together, as synchronized monthly sequences. To generate a base model for subsequent analyses with covariates , return at primary trapping sessions was analyzed with respect to season (monthly variation, April to October) and either year (2008/2009) or site × year (BLB 2008/ SQC 2008/ SCP 2009/ KTH 2009). Models with all possible combinations of additive, interaction and null structures for *p* and *φ* were compared by AICc (Akaike’s information criterion). Once the base model (lowest AICc) was determined, time-specific individual covariates for weight, Gata3^blood^ and weight × Gata3^blood^ were added to the model, with the significance of terms in nested candidate models determined by likelihood ratio tests (LRTs).

**1.5.4. Software**

LMMs and GLMMs were implemented in GenStat version 12.2.03717. PCA and GLMs in Minitab version 16.2.2 and SEM in *R* using the package *Lavaan (S18)*. CJS analyses were conducted in Mark version 6.2.

**SI References S1**

S1. J. A. Jackson *et al.*, *Mol. Ecol.* **20**, 893 (2011).

S2. M. R. O'Donovan, S. Johns, P. Wilcox, *Mutagenesis* **10**, 371 (1995).

S3. A. Schoenemeyer *et al.*, *J. Biol. Chem.* **280**, 17005 (2005).

S4. A. Paun *et al.*, *J. Biol. Chem.* **283**, 14295 (2008).

S5. J. J. Letterio, A. B. Roberts, *Annu. Rev. Immunol.* **16**, 137 (1998).

S6. D. M. Mosser, X. Zhang, *Immunol. Rev.* **226**, 205 (2008).

S7. A. C. Mullen *et al.*, *Science* **292**, 1907 (2001).

S8. D.-H. Zhang, L. Cohn, P. Ray, K. Bottomly, A. Kay, *J. Biol. Chem.* **272**, 21597 (1997).

S9. J. Zhu, H. Yamane, J. Cote-Sierra, L. Guo, W. E. Paul, *Cell Res.* **16**, 3 (2006).

S10. S. Hori, T. Nomura, S. Sakaguchi, *Science* **299**, 1057 (2003).

S11. F. Ramsdell, *Immunity* **19**, 165 (2003).

S12. J. E. Bradley, J. A. Jackson, *Parasitology* **135**, 807 (2008).

S13. S. Burthe *et al.*, *Parasitology* **135**, 309 (2008).

S14. S. Bereczky, A. Martensson, J. P. Gil, A. Farnert, *Am. J. Trop. Med. Hyg.* **72**, 249 (2005).

S15. K. J. Bown, M. Begon, M. Bennett, Z. Woldehiwet, N. H. Ogden, *Emerg. Infect. Dis* **9**, 63 (2003).

S16. S. Telfer *et al.*, *Parasitology* **130**, 661 (2005).

S17. K. J. Bown *et al.*, *Appl. Environ. Microbiol.* **74**, 7118 (2008).

S18. Y. Rosseel, *J. Stat. Softw.* **48**, 1 (2012).

S19. G. C. White, K. P. Burnham, *Bird Stud.* **46**, 120 (1999).

S20. J. D. Lebreton, K. P. Burnham, J. Clobert, D. R. Anderson, *Ecol. Monogr.* **62**, 67 (1992).

S21. R. A. Watkins, S. E. Moshier, W. D. Odell, A. J. Pinter, *J. Protozool.* **38**, 573 (1991).

S22. J. L. Coleman, D. LeVine, C. Thill, C. Kuhlow, J. L. Benach, *J. Infect. Dis.* **192**, 1634 (2005).
